# Dietary salt promotes cognition impairment through GLP-1R/mTOR/p70S6K signaling pathway

**DOI:** 10.1038/s41598-024-57998-9

**Published:** 2024-04-04

**Authors:** Xu Yang, Shu Liu, Chuanling Wang, Haixia Fan, Qian Zou, Yingshuang Pu, Zhiyou Cai

**Affiliations:** 1 Department of Neurology, Affiliated Hospital of Southwest Medical University, Sichuan, 646000 People’s Republic of China; 2 Department of Neurology, Chongqing General Hospital, Chongqing university, No. 118, Xingguang Avenue, Liangjiang New Area, Chongqing, 401147 People’s Republic of China; 3 Chongqing Key Laboratory of Neurodegenerative Diseases, Chongqing No. 312, Zhongshan First Road, Yuzhong District, Chongqing, 400013 People’s Republic of China; 4 Department of Pathophysiology, School of Basic Medicine, Chongqing Medical University, No. 1 Yixueyuan Road, Yuzhong District, Chongqing, 400016 People’s Republic of China; 5 Department of Neurology, Chongqing General Hospital, No. 312 Zhongshan First Road, Yuzhong District, Chongqing, 400013 People’s Republic of China

**Keywords:** Dietary salt, High-salt diet (HSD), Normal diet (ND), Cognition impairment, Tau hyperphosphorylation, Neuroscience, Neurology

## Abstract

Dietary salt has been associated with cognitive impairment in mice, possibly related to damaged synapses and tau hyperphosphorylation. However, the mechanism underlying how dietary salt causes cognitive dysfunction remains unclear. In our study, either a high-salt (8%) or normal diet (0.5%) was used to feed C57BL/6 mice for three months, and N2a cells were cultured in normal medium, NaCl medium (80 mM), or NaCl (80 mM) + Liraglutide (200 nM) medium for 48 h. Cognitive function in mice was assessed using the Morris water maze and shuttle box test, while anxiety was evaluated by the open field test (OPT). Western blotting (WB), immunofluorescence, and immunohistochemistry were utilized to assess the level of Glucagon-like Peptide-1 receptor (GLP-1R) and mTOR/p70S6K pathway. Electron microscope and western blotting were used to evaluate synapse function and tau phosphorylation. Our findings revealed that a high salt diet (HSD) reduced the level of synaptophysin (SYP) and postsynaptic density 95 (PSD95), resulting in significant synaptic damage. Additionally, hyperphosphorylation of tau at different sites was detected. The C57BL/6 mice showed significant impairment in learning and memory function compared to the control group, but HSD did not cause anxiety in the mice. In addition, the level of GLP-1R and autophagy flux decreased in the HSD group, while the level of mTOR/p70S6K was upregulated. Furthermore, liraglutide reversed the autophagy inhibition of N2a treated with NaCl. In summary, our study demonstrates that dietary salt inhibits the GLP-1R/mTOR/p70S6K pathway to inhibit autophagy and induces synaptic dysfunction and tau hyperphosphorylation, eventually impairing cognitive dysfunction.

## Introduction

High-salt diet (HSD) is an independent risk factor for many diseases. In recent years, the association between HSD and conditions such as hypertension, cerebrovascular diseases, and cognitive dysfunction^1^ has been gradually revealed. Studies have shown that the sodium intake in almost all countries exceeds the recommended levels^2^, indicating that a significant portion of the global population is exposed to HSD to varying degrees. Studies have found that HSD impairs brain structure and function by affecting the level of synaptic proteins and microtubule-associated proteins^3^. Additionally, another study showed that HSD can lead to hyperphosphorylation of tau, which is considered an independent risk factor for cognitive impairment^4^.

Glucagon-like peptide-1 receptor (GLP-1R) is a G protein-coupled receptor expressed in many tissues such as the pancreas, brain, kidney, lung, and heart^5^. It is mainly activated by GLP-1. GLP-1R agonists are known for their use in the treatment of diabetes. However, in recent years, more and more studies have found that GLP-1R can be used as a therapeutic target for neurodegenerative diseases such as Alzheimer's disease (AD) and Parkinson's disease (PD)^6–8^. Autophagy is a pathway through which cytoplasmic materials are degraded and recycled^9^ and is activated to maintain cellular homeostasis under conditions of cellular stress, such as oxidative stress, starvation, hypoxia, or exposure to toxic substances^10^. Research has shown that both defective and excessive autophagy can be harmful, and the dysregulation of autophagy is involved in cancer^11^ and neurodegenerative diseases^12,13^. The autophagy pathway is a dynamic process that mainly includes the following steps: formation of the isolation membrane, elongation and maturation of the phagophore to form autophagosomes, autophagosome-lysosome fusion, and lysosome-cargo degradation^14^. Many autophagy-related proteins are involved in the autophagy process. Beclin1 can initiate the formation of autophagosomes, mediate the recruitment of autophagy-related proteins, and promote the formation and maturation of autophagosomes^15^. Microtubule-associated protein light chain 3β, is a crucial macroautophagy protein that is present from autophagosome development to lysosome fusion. There are two kinds of LC3: lipidated LC3-II and soluble LC3-I. Autophagy-related protein 7 (Atg7) initiates the activation of LC3I, which is then transferred to Atg3. The transformation of LC3 from its cleaved form (LC-I) to its conjugated form (LC3II) is regarded as a crucial stage in the creation of autophagosomes^16,17^. The sequestosome 1 gene (SQSTM1), also named p62, is a selective autophagy receptor that selectively integrates into autophagosomes by directly binding to LC3 on the autophagic membrane and is subsequently degraded in autophagosomes. The total cellular expression level of p62 is inversely proportional to autophagic activity^18^. Therefore, markers such as Beclin1, LC3 and P62 were used to reflect the level of autophagy. The mammalian target of rapamycin (mTOR) is a serine/threonine kinase^19^ that serves as a negative regulator upstream of the autophagy pathway. As a molecule downstream of mTOR, the phosphorylation level of ribosomal S6 protein kinase (p70S6K) can reflect the degree of mTOR activation. In previous studies, it has been found that mTOR/p70S6K is closely related to the pathogenesis of AD, and inhibition of the mTOR/p70S6K pathway can weaken tau phosphorylation and oxidative stress in rats^20^.

The relationship between GLP-1R and autophagy has been a subject of research interest. In one study, liraglutide, a GLP-1R agonist, improved renal function in a rat model of chronic renal failure with residual kidney by regulating AMPK/mTOR pathway to promote autophagy^21^. Another study found that GLP-1R promoted functional recovery in acute spinal cord injury by promoting autophagy^22^. Autophagy and GLP-1R are closely related to the pathogenesis of nervous system diseases, but how they affect and participate in the development of cognitive function in the HSD model needs to be further investigated. Based on previous studies, we hypothesize that a long-term HSD regulates autophagic flux by impairing the level of GLP-1R, thereby damaging neurons and synapses, and subsequently leading to cognitive impairment. In this study, we preliminarily explored the changes in GLP-1R and autophagic flux in mice on a long-term HSD. The purpose of our study is to determine the impact of HSD on GLP-1R, mTOR/p70S6K pathway, and tau phosphorylation, hopefully providing valuable insights for future studies.

## Material and methods

### Animals and treatments

We purchased male C57BL/6 mice (9 months old, 25–33 g) from Chongqing Byrness Weil Biotech Co., Ltd. (Chongqing, China). The mice were housed in standard cages at the Animal Center of the Chongqing Key Laboratory of Neurodegenerative Diseases (Chongqing, China), with adequate water and suitable environmental conditions (12 h light/12 h dark cycle at 25 ± 2 °C). Bedding materials were changed on a regular basis. The mice were given one week to adapt to the environment, and after one week, they were randomly divided into a sodium-rich group and a normal diet group, which were provided with 8% and 0.4% NaCl diet (Tengxin Biotechnology Co., Ltd., Chongqing, China) for 3 months, respectively. All other feeding conditions were the same. The initial body weight of the mice was recorded at the time of purchase and then measured monthly. We also recorded their daily intake of water and food and calculated the average water intake and food intake of each C57BL/6 mouse after three months for statistical analysis. All experimental protocols were approved by the Research Ethics Committee of Chongqing Medical University. All methods were carried out in accordance with the American Veterinary Medical Association (AVMA) Guidelines for the Euthanasia of Animals (2020) and The National Institutes of Health Guide for the Care and Use of Laboratory Animals. All methods are reported in accordance with ARRIVE guidelines. After three months of feeding, the mice underwent the open field test (OFT), the Morris water maze (MWM), and the shuttle box test (SBT). After the behavioral experiment, the mice were euthanized using cervical dislocation for immunohistochemistry, immunofluorescence, electron microscopy, and Western blotting. The brain tissue used for different experiments was processed differently. The brains of the mice were deeply anesthetized with isoflurane and fixed by transcardiac perfusion, and then the intact brains were removed for immunohistochemistry, immunofluorescence, and electron microscopy. The brains of the mice were removed immediately after the mice were euthanized by cervical dislocation and immediately placed at − 80 °C for further Western blotting.

### Cell culture and treatments

To investigate the impact of an HS diet on neuronal cells, we established a high-salt cell model using the Neuro-2a (N2a) cell line, which was provided by Procell Life Science & Technology Co., Ltd. (Wuhan, China) The N2a cells were maintained in a humidified incubator at 37 °C and 5% CO_2_ and were cultured in Dulbecco's modified Eagle's medium nutrient mixture F-12 (DMEM-F12, Gibco, Carlsbad, CA, USA) with 10% fetal bovine serum (FBS; Invitrogen, Irvine, CA, USA) and antibiotics (100 U/mL penicillin and 100 μg/mL streptomycin)^18^. N2a cells were treated with the medium supplemented with NaCl (adding 80 mM NaCl) for 48 h to establish the HS N2a cell model^23^, and no additional treatment was performed in the ND group. The cells were subsequently treated with liraglutide (200 nM; HY-P0014, MCE) + NaCl (80 mM) for 48 h to study the role of GLP-1R.

### Behavioral experiment

#### Morris water maze (MWM)

MWM was conducted to examine mice’s memory and spatial learning and it was performed after three months of a high-salt diet (HSD, n = 10/group) and a normal diet (ND, n = 10/group). We divided the circular pool (diameter 1.2 m, depth 0.4 m, 25 ± 1 °C) into four quadrants (I, II, III, IV), and an 8 cm circular platform was placed inside the northeast quadrant (quadrant I), which was 1 cm lower than the water surface. A camera was positioned above the maze's center to capture the mice's movements and transmitted the data to ANY-maze video tracking software (Stoelting Co., Wood Dale, IL, USA). The C57BL/6 mice were placed into the maze facing away from the platform, each from different quadrants, and swam freely for 1 min. After reaching the platform, the mice were allowed 5 s to stay on the platform. If a mice didn’t enter the platform within 1 min, it was guided to the platform and allowed to stay on the platform for 30 s (the mice were removed and dried immediately after each round). We removed the platform on the sixth day. The mice were allowed to enter the maze from quadrant III, and they were given 60 s to swim. We used the ANY-maze video tracking software (Stoelting Co., Wood Dale, USA) to log trajectories and all data including total distance, swim time in different quadrants, and number of entries to the platform.

#### Shuttle box test (SBT; Passive avoidance test (PAT))

SBT was conducted using a dark avoidance shuttle tester (SCIMON Intelligent Technology Co., Ltd), so it's also called the Passive Avoidance Test (PAT). Animals were allowed to move freely in the test box for 5 min to eliminate exploration reflexes, and then the mice were put in the electric shock area of the shuttle test box, where they were exposed to 10 s of light and a buzzing sound, followed by an electric shock (0.4 mA, 50 Hz) for 5 s. Active avoidance response was defined as the mouse escaping to the safety zone within 10 s after the light was turned on, and passive avoidance response was defined as the mouse escaping to the safety zone after the electric shock was given. Each training session lasted for 20 s, and a total of 50 training sessions were performed. After the training was completed, the experiment was conducted (total test time: 5 min, shock time: 5 s, prompt time: 10 s, interval time: 5 s, number of cycles: 30), and the data of escape latency, number of active escapes, and number of passive escapes were recorded by the dark avoidance shuttle tester.

#### Open-field test (OFT)

The OFT was conducted in a quiet environment to assess anxiety in the mice. The animals were put on the box’s bottom surface, then video recording and timing were started simultaneously. After 3 min of observation, the video recording was stopped, and the walls and floor of the open field box were cleaned using alcohol to remove the remaining information about the previous one (such as the animal's feces, urine, and smell) to prevent them from affecting the results of the next one. ANY-maze video software (Stoelting Co., Wood Dale, USA) was used to transcribe data (total distance, center time, number of entries in center) and trajectories.

### Transmission electron microscope (TEM)

After the behavioral experiments, the brains of C57BL/6 were deeply anesthetized with isoflurane and fixed by transcardiac perfusion, and then the brains were extracted. The hippocampus was then quickly isolated, and dehydrated by the these reagents in the following order: 3% buffered glutaraldehyde (4 h), 1% osmium tetroxide (GP18456, Leica, 2 h), acetone (50%, 70%, 80%, and 90%), and 100% acetone (15 min, twice). Dehydrated hippocampal tissues were then encapsulated in Epox 812 to obtain solid embedding blocks, then sliced into sections (50 nm) using an ultramicroscopy (EM UC7, Leica), which was dyed with uranyl acetate (45 min) and lead citrate (15 min). Finally, the neurons were observed using Transmission Electron Microscope (JEM-1400FLASH, Japan).

### Western blotting

After the behavioral experiments, the C57BL/6 mice were euthanized, and the hippocampus and the cortex were rapidly separated, then placed in separate tubes, and stored at − 80 °C. N2a cells for protein extraction were treated with NaCl (80 mM) or liraglutide (200 nM) for 48 h, and the concentration of extracted proteins was analyzed using a BCA protein analysis kit (Beyotime Biotechnology, Shanghai, China). To analyze the level of proteins with different molecular weights, we loaded the proteins (50 µg) from mice brains or cells onto 6%, 10%, or 12.5% SDS-PAGE gels for electrophoresis. To ensure consistent protein loading in each Western blot experiment, we used an internal reference protein as the evaluation index in each experiment. When the internal reference was uniform across groups, we considered the protein loading in each well to be consistent. Subsequently, the proteins on the gels were transferred to PVDF membranes (Bio-Rad, Hercules, USA), then blocked with TBST containing 5% milk for 2 h, and incubated at 4 °C overnight with the following primary antibodies: rabbit anti-GLP-1R (1:500, cat.97308, Novus Biologicals, USA), rabbit anti-beclin1 (1:1000, cat. #3495, Cell Signaling Technology, USA), rabbit anti-SQSTM1/p62 (1:1000, cat.#23214, Cell Signaling Technology, USA), rabbit anti-LC3A/B (1:1000, cat.#12741, Cell Signaling Technology, USA), rabbit anti-mTOR(1:1000, cat.#2983, Cell Signaling Technology, USA), rabbit anti-phospho-mTOR (Ser2448, 1:1000, cat.#5536, Cell Signaling Technology, USA), rabbit anti-p70S6 Kinase (1:1000, cat.#34475, Cell Signaling Technology, USA), rabbit anti-phospho-p70S6 Kinase (1:1000, cat.#9234,Cell Signaling Technology, USA), rabbit anti-PSD95 (1:1000, Cell Signaling Technology, USA), Mouse anti-SYP (1:2000, sc-17750, Santa Cruz, USA), rabbit anti-MAP2 (1:500, bs-1369R, Bioss Biotechnology Co., Ltd, China), mouse anti-Tau46 (1:1000, cat. #4019, Cell Signaling Technology); rabbit anti-phospho-tau Thr205 (1:1000, cat. #49561, Cell Signaling Technology, USA); rabbit anti-phospho-tau Thr181 (1:1000, cat. #12885, Cell Signaling Technology, USA); rabbit anti-phospho-tau Ser404 (1:1000, cat. #20194; Cell Signaling Technology, USA); mouse anti-GAPDH (1:1000, cat. AF0006, Beyotime Biotechnology); mouse anti-β-actin (1:1000; cat. AF5003; Beyotime Biotechnology). Then the membranes were shocked with TBST (3 × 10 min) and incubated with secondary antibodies (anti-mouse IgG or anti-rabbit IgG, Beyotime Biotechnology) at room temperature for 1 h. Ultimately, an ECL chemiluminescent system (Tanon-5200Multi, Shanghai, China) was used to expose the aim protein, and ImageJ 1.8.0 software (NIH) was employed for calculating the gray value of the target protein bands.

### Immunofluorescence staining

Mice’s brains were fixed with 4% paraformaldehyde for 48 h before paraffin embedding and then sliced into 20 µm sections utilizing the Rotary Microtome (HM 340E, Thermo Scientific). Antigen retrieval was performed by using a citric acid solution to the sections (high fire: 5 min, medium fire: 15 min). Following antigen retrieval, the sections were blocked with 5% goat serum (room temperature, 30 min) and then incubated overnight at 4 °C with the primary antibodies LC3A/B (1:100, cat. # 12741, Cell Signaling Technology, USA) and GLP-1R (1:100, cat. 97308, Novus Biologicals, USA). The next day, the sections were taken out of the 4 °C refrigerator to rewarm, then treated for 60 min at ambient temperature with secondary antibodies (Alexa Fluor 488-coupled antibody (ZSGB-BIO, Beijing, China)) before being stained with DAPI (Beyotime Biotechnology) and ultimately analyzed with a NEXCOPE microscope (NE900, Ningbo Yongxin Optics Ltd., Ningbo, China). ImageJ software was used to quantify the level of LC3A/B and GLP-1R. The sample size was n = 3/group.

### Immunohistochemical staining

The brains of the animals were processed into paraffin sections using a rotating slicer (HM 340E, Thermo Scientific). The sections were then dehydrated and deparaffinized. Antigen retrieval was conducted using a citric acid solution (5 min of intense fire, 15 min of medium fire). Next, the sections were blocked for 30 min at room temperature with 5% goat serum before being incubated overnight with the primary antibodies GLP-1R (1:200, cat. 97308, Novus Biologicals, USA) and LC3B (1:100, cat.bs-2912R, Bioss). The next day, the sections were brought to room temperature and then incubated with secondary antibodies (goat anti-rabbit/mouse IgG) for 20 min. The sections were then stained using DAB and hematoxylin. Finally, photos were taken by the NEXCOPE microscope after the sections were blown dry and sealed with neutral balsam. ImageJ software was utilized to analyze the level of LC3B and GLP-1R. The sample size was n = 3/group.

### Statistical analysis

GraphPad Prism Software version 9.5.1(GraphPad Software, San Diego, CA, USA) was used for the statistical analysis of the experimental data. All data are presented as mean ± standard deviation (SD). One-way analyses of variance (ANOVA) and independent-sample t-tests were utilized for analysis, *p* < 0.5 was deemed statistically significant.

### Ethical approval and consent to participate

The Research Ethics Committee of Chongqing Medical University approved the ethical guidelines for animal research. The study is reported in accordance with ARRIVE guidelines.

## Results

### HSD promotes cognitive impairment in C57BL/6 mice

**Figure 1:**
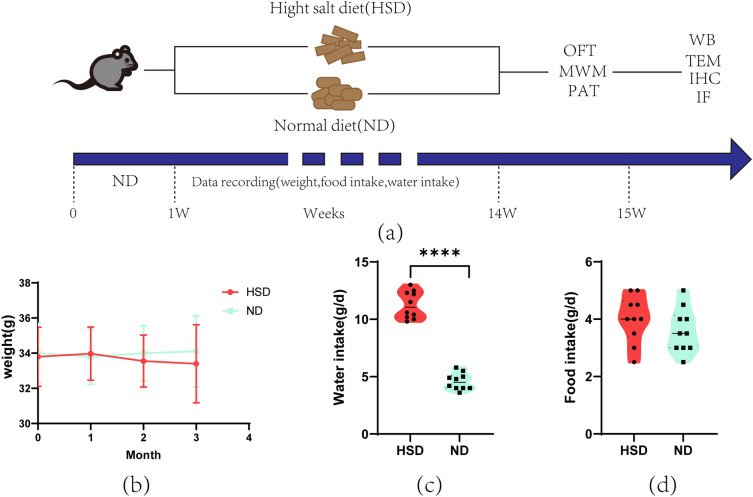
General characteristics of C57BL/6 mice fed a high-salt diet or a normal diet. (**a**) C57BL/6 mice (9 months old) were fed HSD or ND for 3 months. (**b**) and (**d**) weight and food intake were not significantly different between groups, n = 10/group, two-sample *t*-test. (**c**) Water intake was significantly different between groups, n = 10/group, two-sample *t*-test. Data are shown as the mean ± SD. ****p < 0.001. HSD: high-salt diet; ND: normal diet; OFT: open field test; MWM: Morris water maze; PAT: passive avoidance test; TEM: transmission electron microscopy; WB: western blotting; IHC: Immunofluorescence; IF: Immunofluorescence.

Throughout the three months of group feeding (Fig. 1a), we found that the body weight and food intake of mice showed no significant difference between HSD and ND, while the water intake in HSD was significantly higher than that in the ND group (Fig. 1b–d).

**Figure 2:**
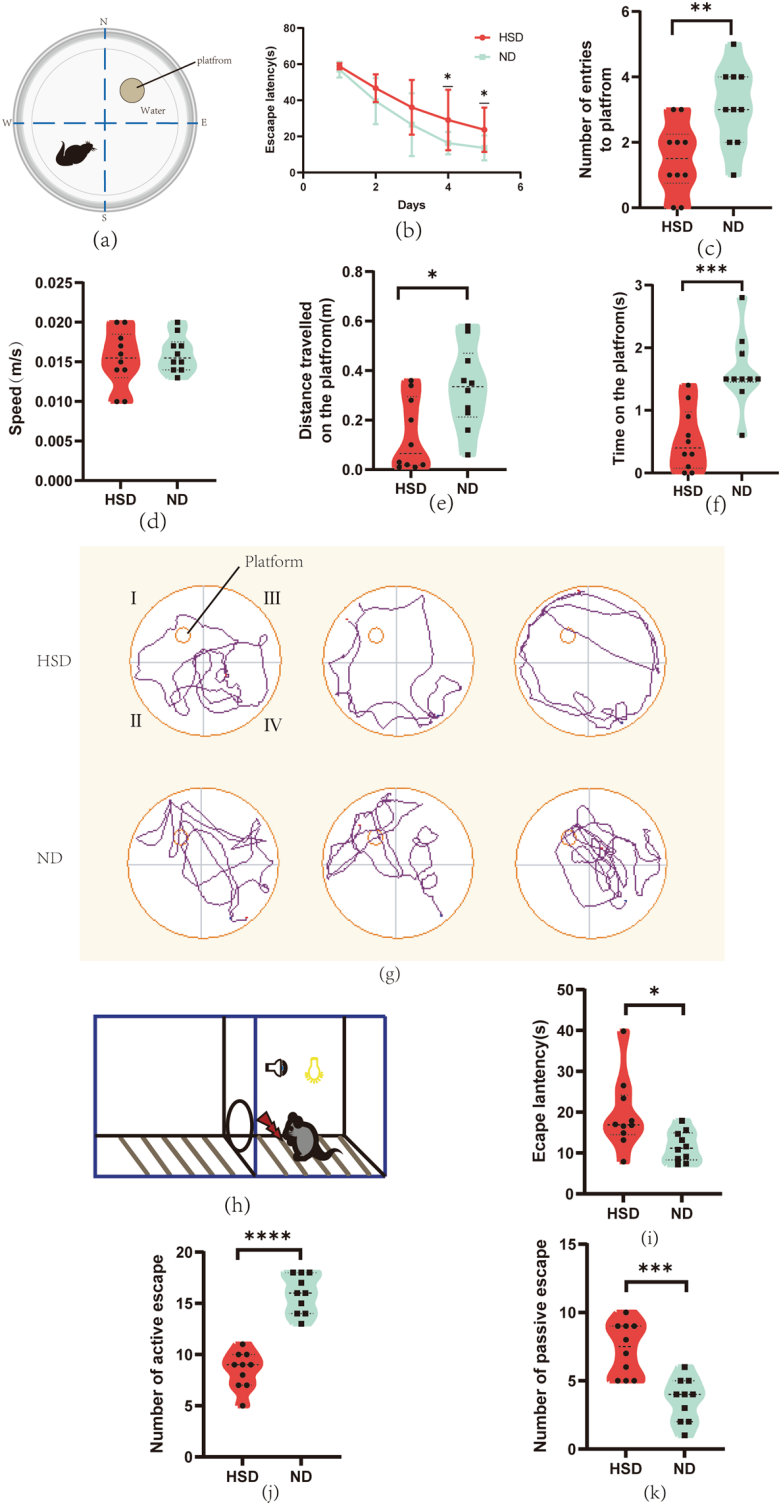
Dietary salt damages spatial learning and memory in C57BL/6 mice. Morris water maze: (**a**) Schematic diagram of Morris water maze in C57BL/6 mice. The C57BL/6 mice were divided into two groups, the HSD group was fed a high-salt diet for 3 months; the ND group was fed a normal diet for 3 months. Compared to the ND group, HSD led to significant changes in average escape latency (**b**), number of entries to platform (**c**), distance traveled in the platform (**e**), and time on the platform (**f**). Speed (**d**) was not significantly different between groups. Swimming traces of mice in the probe trial are presented in (**g**). Shuttle Box test: (**h**) Schematic diagram of Shuttle Box in C57BL/6 mice. In the shuttle box test, the escape latency (**i**), number of active escape (**j**), and number of passive escape (**k**) of the two groups of mice all showed significant statistical differences. n = 10/group, two-sample *t*-test. Mean ± SD, *p < 0.05, **p < 0.01, ***p < 0.001. HSD: high-salt diet; ND: normal diet.

To study the impact of dietary salt on the cognitive function of mice, we conducted MWM (Fig. 2a) and SBT (Fig. 2h) to assess their spatial memory and learning function. The escape latency of the two groups of mice in MWM appeared to decrease in a time-dependent manner, and the escape latency of the HSD group was longer than that of the ND group on the fourth and fifth days (Fig. 2b). In contrast, the number of entries to the platform (Fig. 2c), distance traveled on the platform (Fig. 2e), and time on the platform (Fig. 2f) were significantly lower in HSD mice than in ND. Notably, there was no significant difference in swimming speed between the two groups (Fig. 2d). This ruled out the possibility that these differences between groups were attributable to differences in the mice's physical mobility. The trajectories of two groups in the MWM are shown in Figure (Fig. 2g). The SBT showed that the escape latency of HSD was longer than ND (Fig. 2i), the number of the active escape of HSD was lower than ND (Fig. 2j), while the number of the passive escape was higher (Fig. 2k). In conclusion, these results show that the HS diet impairs spatial memory and learning ability in mice.

### Dietary salt does not cause anxiety in C57BL/6 mice

**Figure 3:**
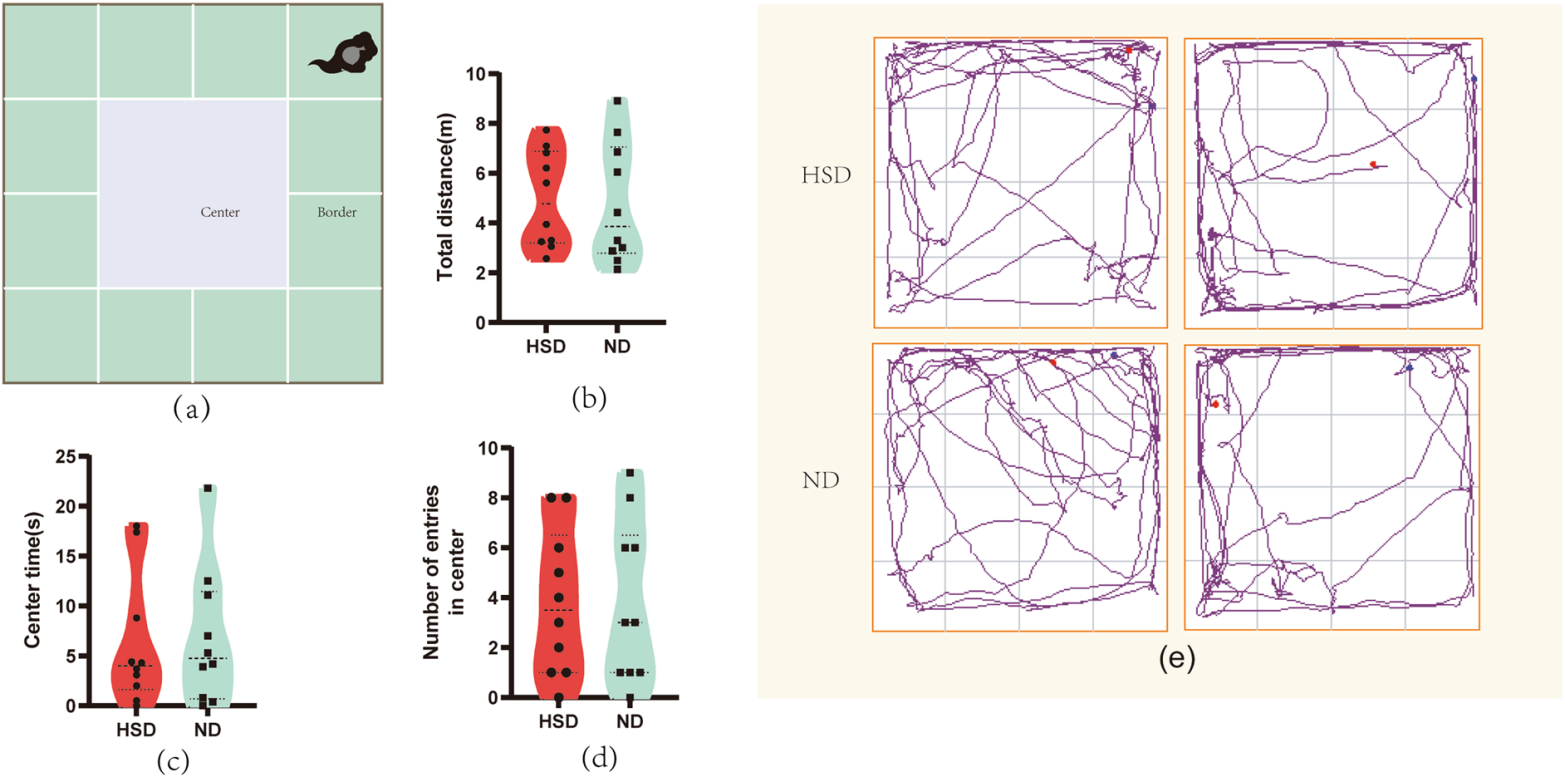
Dietary salt does not cause anxiety in C57BL/6 mice. (**a**) Schematic diagram of Open field test in C57BL/6 mice. There were no significant differences between the HSD group and the ND group in the total distance (**b**), center time (**c**), and number of entries in center (**d**). The trajectory of exploration of mice in the probe trial is presented in (**e**). n = 10/group, two-sample *t*-test. Mean ± SD. HSD: high-salt diet; ND: normal diet.

We used the OFT (Fig. 3a) to assess the impact of the HS diet on anxiety in mice, and the data are shown in Fig. 3 (Fig. 3b–e). The data show that the total distance within 3 min, the time spent in the center area, and the number of entries into the center were similar between the two groups. This indicates that the HS diet did not induce anxiety in mice.

### HSD inhibited autophagy via GLP-1R/mTOR/p70S6K pathway

**Figure 4:**
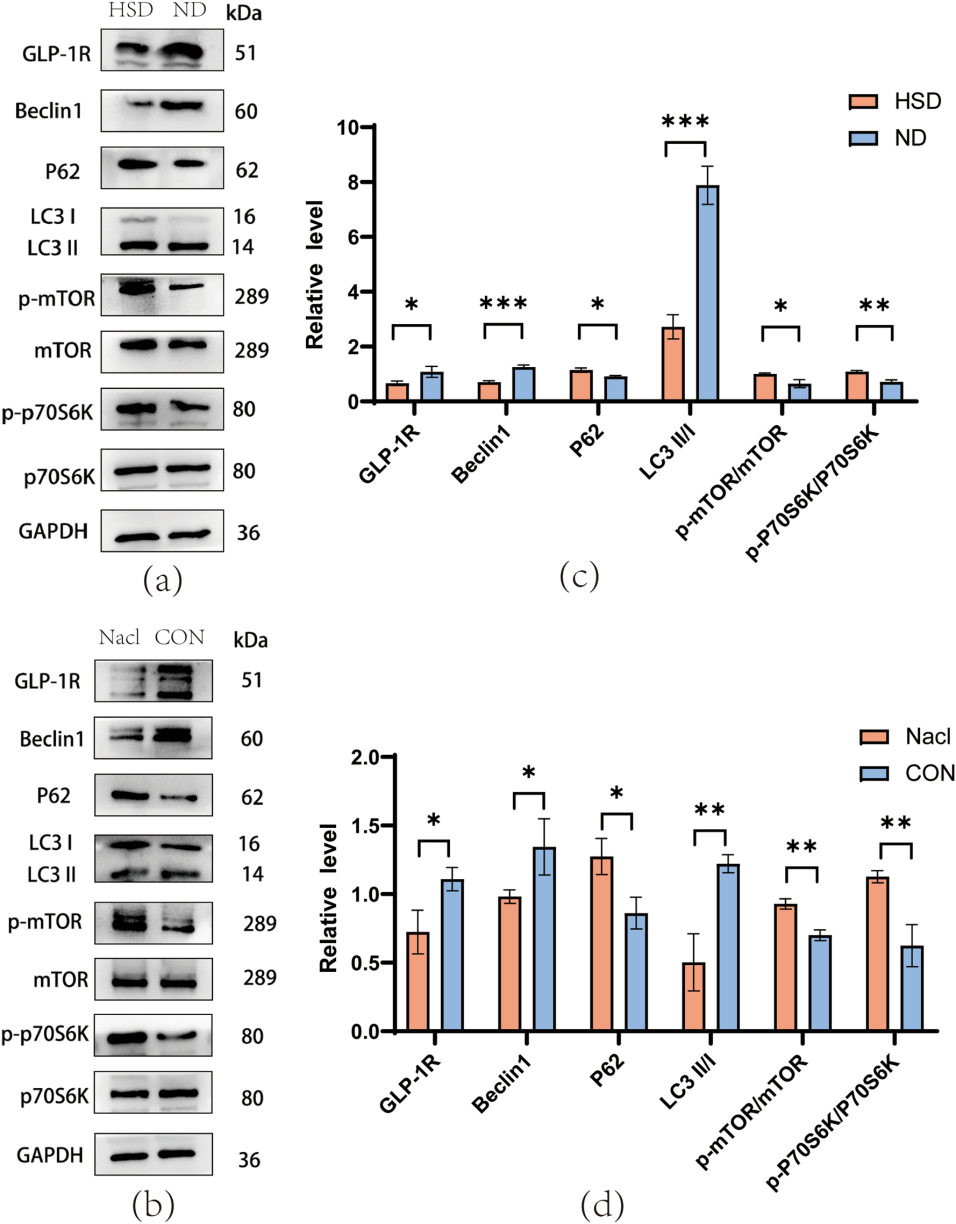
Level of proteins related to GLP-1R and autophagy. (**a**, **b**) The western blotting indicated that HSD reduced the level of GLP-1R, Beclin1, and LC3II and increased the level of p62, p-mTOR, and p-p70S6K in the brains of C57BL/6 mice (n = 4/group, two-sample t-test). (**c**, **d**) The level of proteins related to GLP-1R and autophagy in N2a cell. This level profile was in line with the in vivo results (two-sample *t*-test). Relative level of proteins normalized to GAPDH. Mean ± SD, *p < 0.05, **p < 0.01, and ***p < 0.001. ND: normal diet; HSD: high-salt diet; CON: control; GAPDH: glyceraldehyde-3-phosphate dehydrogenase.

**Figure 5:**
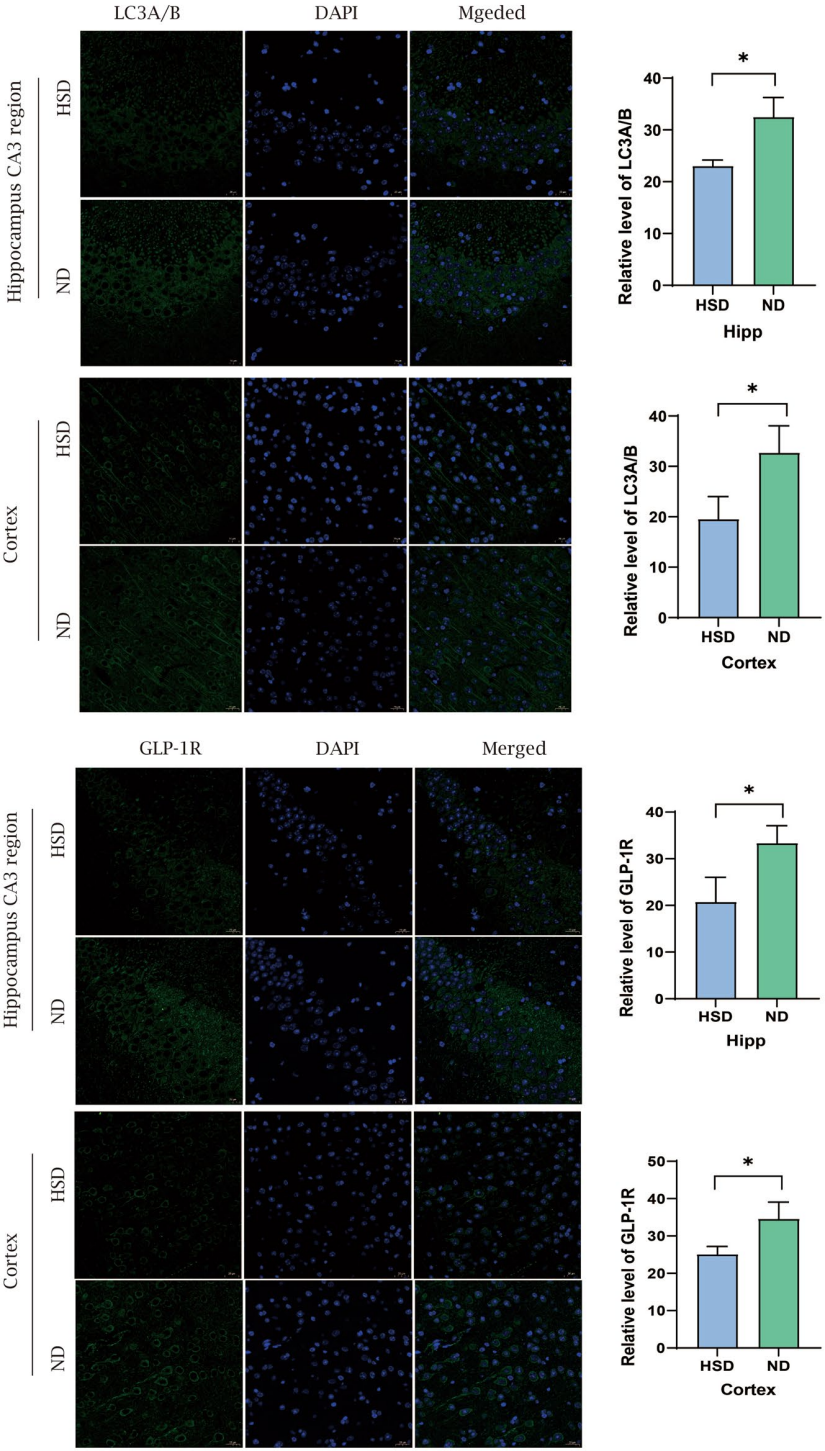
Immunofluorescence staining of GLP-1R and LC3A/B. Immunofluorescence observed the level of GLP-1R and LC3A/B of the hippocampus and cortex (n = 3/group, two-sample *t*-test). Mean ± SD; *p < 0.05. Scale bar = 20 µm. ND: Normal diet; HSD: high-salt diet; Hipp: hippocampus.

**Figure 6:**
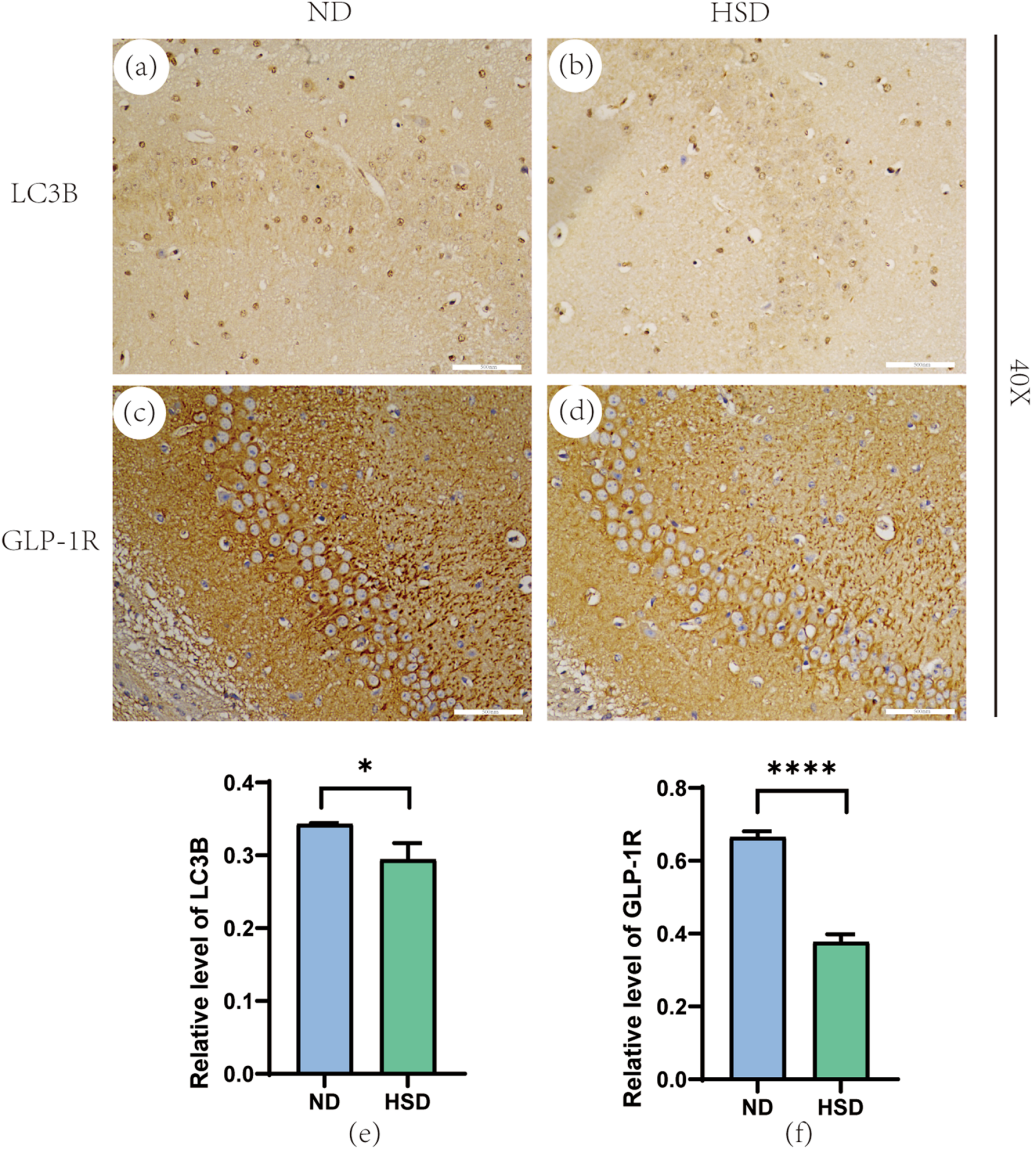
Hippocampal immunohistochemical staining of GLP-1R and LC3B. (**a**, **b**, **e**) shows the level of LC3B in region CA3 of the hippocampus, and there was a clear difference in the level of the two groups. (**c**, **d**, **f**) shows the level of GLP-1R in region CA3 of the hippocampus, and GLP-1R was significantly lower in the HSD group than in the ND group. (n = 3/group, two-sample *t*-test). Mean ± SD; *p < 0.05, ****p < 0.0001. Scale bar = 500 nm. ND: Normal diet; HSD: high-salt diet.

To investigate the relationship between HS diet and autophagy, we established both in vivo and in vitro high-salt models and used Western blotting (WB) to analyze proteins related to GLP-1R/mTOR/p70S6K pathway (Fig. 4). The WB results showed that compared with the ND group, the level of GLP-1R of the HSD decreased, and the level of autophagy-related proteins Beclin1 and LC3II/LC3I decreased, while the level of P62 increased in the HSD group. In addition, we examined the level of mTOR pathway-related proteins and found that the level of phosphorylated mTOR (p-mTOR) and phosphorylated p70S6K (p-p70S6K) in the HSD group was higher compared to the ND group (Fig. 4a, b), indicating that the HS diet inhibits autophagy in the C57BL/6 mice's brain tissue through GLP-1R/mTOR/P70S6K pathway. In the in vitro model, N2a cells treated with NaCl also exhibited reduced levels of GLP-1R, Beclin1, and LC3 II/I but with elevated levels of P62, p-mTOR and p-p70S6K compared to the ND group (Fig. 4c, d). Moreover, our immunofluorescence results demonstrated that the GLP-1R and LC3 A/B fluorescence intensity in the cortex and hippocampus of the HSD group was lower than that the ND group (Fig. 5), consistent with the Western blotting findings. Additionally, immunohistochemistry revealed that the HSD group had lower levels of GLP-1R and LC3B activation in the hippocampus as compared to the ND group (Fig. 6). In conclusion, high salt diet inhibits autophagy through GLP-1R/mTOR/p70S6K pathway.

**Figure 7:**
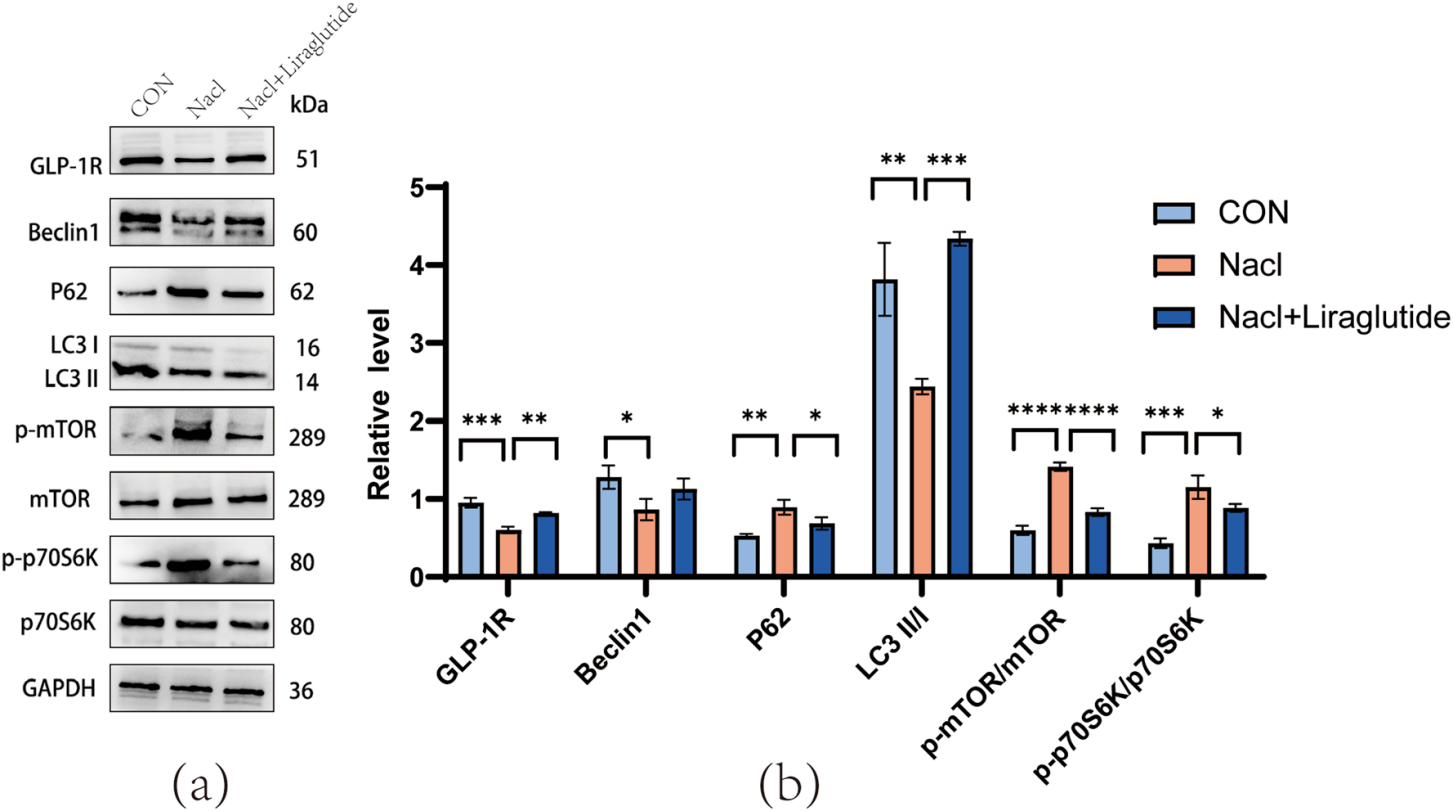
Level of autophagy-related proteins after liraglutide treatment. (**a**, **b**) The level of proteins related to autophagy in N2a cells is treated with Liraglutide medium (200 nM). Liraglutide reversed the inhibition of GLP-1R and autophagy induced by high salt, compared to the HSD group, NaCl + Liraglutide group increased the level of GLP-1R, LC3AB II/I, and reduced the level of p62, p-mTOR, and p-p70S6K. (one-way ANOVA). Relative level of proteins normalized to GAPD. Mean ± SD, *p < 0.05, **p < 0.01, ***p < 0.001, and ****p < 0.0001. ND: Normal diet; HSD: high-salt diet; CON: control.

To further investigate the relationship between GLP-1R and mTOR/p70S6K, we treated N2a cells with GLP-1R agonist liraglutide, and assessed related proteins using WB. The WB results revealed that liraglutide reversed the level in autophagy-related proteins LC3II/LC3I, P62, p-mTOR and p-p70S6K caused by HSD (Fig. 7a, b), but liraglutide did not affect the level of Beclin1. This finding suggests that activation of GLP-1R reverses autophagy inhibition caused by HSD.

### HSD elevated tau phosphorylation in N2a cells and C57BL/6 mice

**Figure 8:**
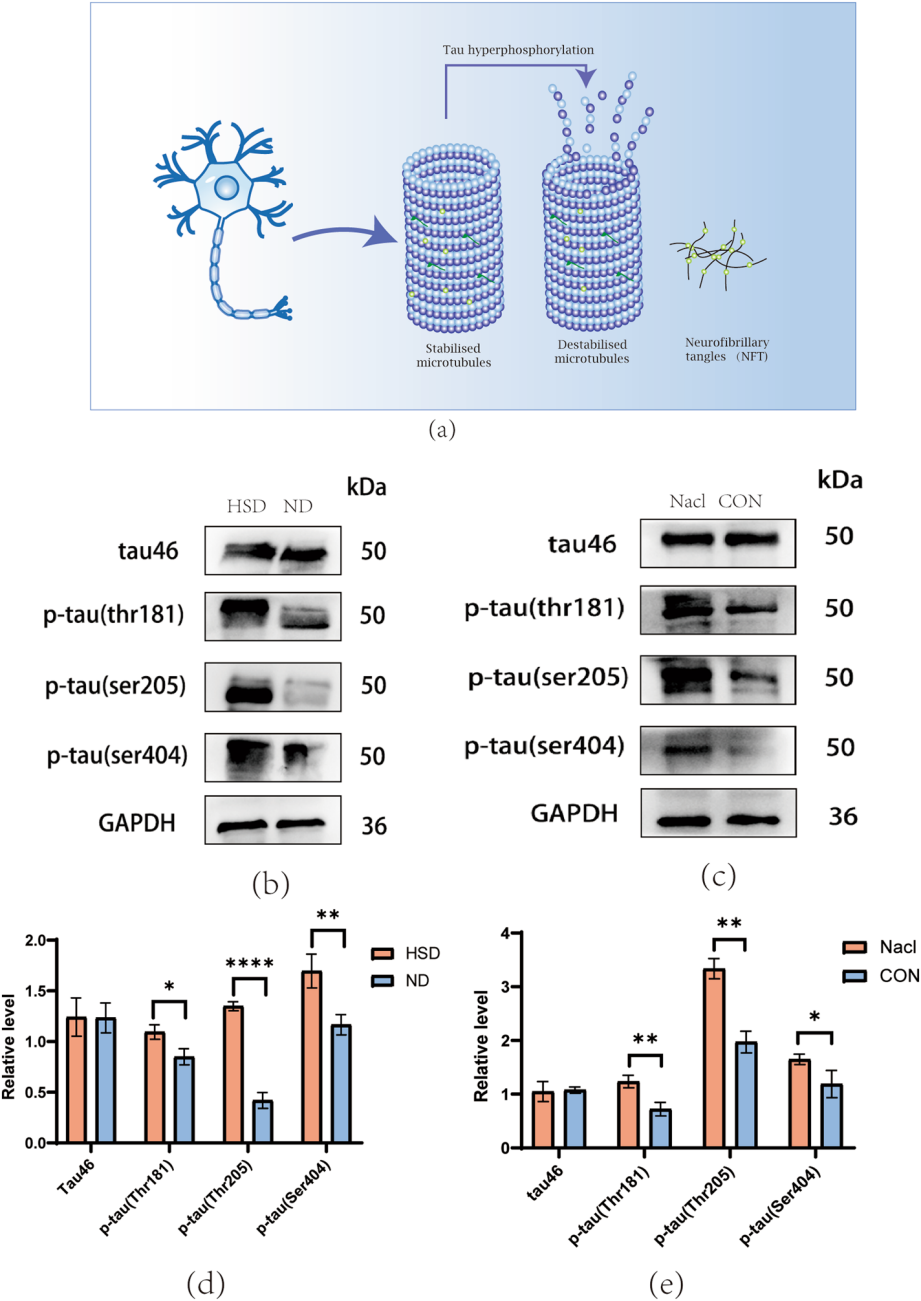
A high salt diet induces tau protein hyperphosphorylation. (**a**) Schematic diagram of tau hyperphosphorylation. (**b**, **d**) HS diet generated an increase in p-tau (Thr181, Thr205, Ser404) in mice (n = 3/group, two-sample *t*-test). (**c**, **e**) HS diet generated an increase in p-tau (Thr181, Thr205, Ser404) in N2a cell (two-sample *t*-test). Relative level of proteins normalized to GAPDH. Mean ± SD; *p < 0.05, **p < 0.01, ***p < 0.001, and ****p < 0.0001. ND: Normal diet; HSD: high-salt diet; CON: control; TNF: neurofibrillary tangles.

WB was performed to investigate how HSD affected tau46 (total tau) and p-tau, and the experiments revealed that the HSD group had significantly higher levels of p-tau(thr181), p-tau(thr205), and p-tau(ser404) level than the ND group in both in vivo and in vitro models (Fig. 8).

### Excessive salt damages neurons and destroys synapses

**Figure 9:**
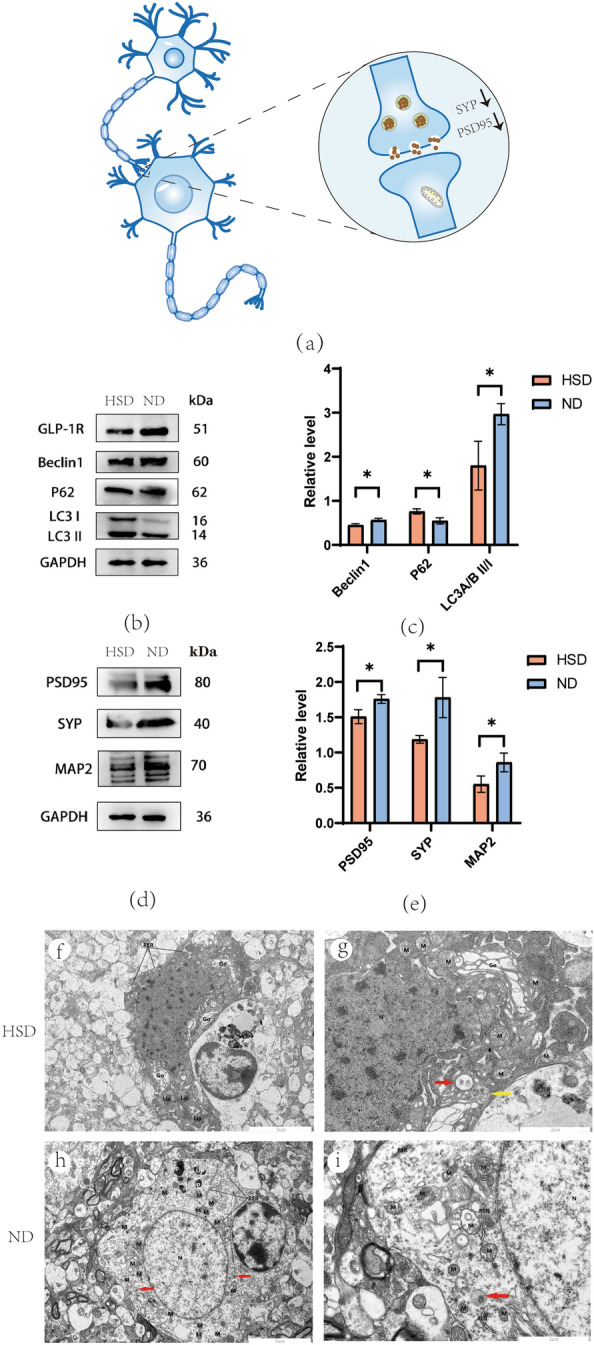
Level of synaptic proteins and autophagy in the hippocampus. (**a**) Diagrammatic sketch of neurons and synapse. (**b**, **c**) Dietary salt damaged GLP-1R and autophagy flux. (**d**, **e**) Compared with the ND group, the high-salt diet decreased the level of synapse-associated proteins. (**f**, **g**) In the HSD group, the neuronal cells were slightly pyknotic, the cell membrane was intact, and the electron density of the intracellular matrix was slightly increased and uniform; the number of organelles was acceptable, and most of them were swollen. The nuclei (N) were irregularly shaped with intact nuclear membranes. A small number of autolysosomes (red arrows) were observed. (**h**, **i**) In the ND group, the neurons were intact with elliptic nuclei, evenly distributed chromatin and intact nuclear membrane. A small number of autolysosomes (red arrows) were observed. N: nucleus; M: mitochondria; RER: rough endoplasmic reticulum; Go: Golgi body; Lip: Lipofuscin. Relative level of proteins normalized to GAPDH (n = 3/group, two-sample t-test). Data are expressed as the mean ± SD; *p < 0.05. Scale bar = 5 µm and 2 µm. HSD: High-salt diet; ND: normal diet; PSD95: postsynaptic density protein 95; SYP: synaptophysin.

Compared to the ND group, TEM showed that the high-salt diet damaged neurons. In the HSD group, the electron density of the intracellular matrix was uniformly enhanced and slightly pyknotic, the cell membrane of neuronal cells was unbroken; the number of organelles was acceptable, and most of them were swollen; and the nuclei (N) were irregularly shaped with intact nuclear membranes. The neurons in the ND group had intact nuclear membranes, elliptic nuclei, and equally dispersed chromatin (Fig. 9f–i).

We also performed WB to examine the level of related proteins of the hippocampus in C57BL/6 mice (Fig. 9b–e). We found that the autophagy inhibition caused by HSD in mice also drew the same conclusion in the hippocampus. The activation of Beclin1 and LC3II/I in the hippocampus of the HSD group was down-regulated, while P62 was up-regulated (Fig. 9b, c). In addition, synapse-associated proteins and microtubule-associated proteins were also examined, and we found that compared with the control group, the level of PSD95, SYP, and MAP2 of the HSD group was downregulated (Fig. 9a, d, e), suggesting that the long-term salt intake damaged synapses and neurons. This result is consistent with the MWM and SBT conclusions that a high-salt diet leads to impaired cognitive function.

## Discussion

In this study, we found that the HS diet did not significantly change mice’s food intake and body weight, but it did lead to a notable increase in water intake in the HSD group. We also found that in the WMW, the escape latency in the HSD group was longer than that in the control one, while the time spent on the platform, the number of entries to the platform, and the distance traveled on the platform were less than those in the control group. In the SBT, the HSD group also had fewer active escapes than the ND group. These findings demonstrate that dietary salt leads to cognitive dysfunction in C57BL/6 mice. It’s worth-noting that in the OFT, there were no statistically significant differences in the time and number of visits to the central area between the two groups, suggesting that a chronic HS diet does not induce anxiety in C57BL/6 mice.

We also investigated the effects of the HS diet on synaptic function and tau phosphorylation. Our in vivo and in vitro experiments showed that the level of tau phosphorylation (thr181, ser205, ser404) had significant differences, but there was no significant difference in total tau (tau46). Tau is a microtubule-associated protein^24^ that plays an important role in normal physiological processes; however, tau hyperphosphorylation leads to nerve fiber tangles (NFTs), which are involved in the pathophysiological mechanism of the condition of neurodegeneration such as AD^25,26^. We also found that postsynaptic density 95 (PSD95), synaptophysin (SYP) and MAP2 decreased substantially in the HSD group compared to the control group, regardless of whether it was long-term high-salt diet C57BL/6 mice or N2A cell model treated with NaCl. In our study, we conducted Western blotting to assess the level of PSD95, MAP2, and SYP to study the synaptic and neuronal integrity of the hippocampus. PSD95 is a postsynaptic protein that promotes synaptic maturation and has a significant impact on synaptic stability, strength, and plasticity^26^. Research has found a decrease in PSD95 in dementia with PD, dementia with Lewy bodies (DLB), and AD^27^. Synaptophysin (SYP) is a rich synaptic vesicle membrane protein that participates in synaptic vesicle exocytosis and represents synaptic plasticity^28^. MAP2 is a microtubule-associated protein primarily found in the cell body and dendrites of neurons and participates in neuronal growth and synaptic plasticity^29^. Therefore, the decrease of PSD95 and SYP represents the damage to synaptic function, and MAP2 represents the damage to neurons. Our experimental data are consistent with previous studies that tau hyperphosphorylation not only induces neuronal disorders but also is related to synaptic loss^25,30^. Our study shows that an HS diet leads to decreased level of MAP2. Recent research shows that MAP2 can suppress the fibrosis of tau and become a potential therapeutic target for tau-related diseases^31^. Therefore, we speculate that MAP2 impairment under the HS diet may also become one of the mechanisms underlying cognitive dysfunction.

Much attention has been paid to the specific mechanism contributing to synaptic disorders caused by tau hyperphosphorylation. It has been reported that abnormal tau phosphorylation affects the transport of glutamate receptor subunits to postsynaptic density, resulting in reduced mitochondrial-dependent ATP production, eventually causing synaptic loss^32,33^. It has also been reported that abnormal tau phosphorylation can lead to errors in the position of tau at post-synaptic sites, and the possible mechanism is that hyperphosphorylated tau promotes the dissociation of tau/Fyn/PSD95 complex, and eventually induces long-term depression (LTD) and curbs Long-term Potentiation (LTP)^34^. However, our current study lacks sufficient evidence to identify the exact mechanism of synaptic loss and tau hyperphosphorylation under the HS diet. Excessive dietary salt intake causes neuroinflammation and oxidative stress in the brain^35^. Previous studies have identified persistent and increased inflammation in glial cells and neurons as a key cellular driver and regulator of tau pathological deterioration^36^, as well as oxidative stress-induced hyperphosphorylation of tau^37,38^. We therefore hypothesized that a high-salt diet might lead to tau hyperphosphorylation through neuroinflammation and oxidative stress. Therefore, further experimental evidence is needed.

Although our study did not demonstrate the exact mechanism of tau hyperphosphorylation and synapse loss, it is clear that a long-term HS diet can contribute to cognitive impairment. We also found that the HS diet inhibited the level of GLP-1R and autophagy flux in C57BL/6 mice. Western blotting showed that GLP-1R, Beclin1 and LC3II/I of the HSD group were lower than those in the ND group, while p62, p-mTOR, and p-p70S6K in the HSD group were higher than those in the ND group. To validate our in vivo experiment, we used NaCl to establish an in vitro HSD model, and the results were consistent with the in vivo experiment. In our study, Of course, the exact mechanism still needs further experimental demonstration.

A previous study found that in diabetes-related cognitive impairment, liraglutide ameliorated cognitive function by activating the autophagy pathway^39^. To further explore the relationship between GLP-1R and mTOR, we used GLP-1R agonist liraglutide to treat N2a cells added with NaCl. The results showed that the inhibition of autophagy induced by dietary salt was reversed by treatment with liraglutide, which activated autophagy by inhibiting mTOR/p70S6K pathway. A high-salt diet led to downregulation of levels of GLP-1R, a member of the G-protein coupled receptor (GPCR) family, which is known to activate downstream pathways such as phosphatidylinositol-3 kinase/PKC, cAMP/guanine-nucleotide exchange factor (Epac), and cAMP/protein kinase A (PKA)^40^. Studies have shown that cAMP has an inhibitory effect on mTOR activation^41^, we hypothesized that a HS diet down-regulates GLP-1R levels to activate the mTOR pathway by inhibiting the downstream pathway of G protein-coupled receptors. In recent years, the neuroprotective effect of GLP-1R has garnered increasing attention from researchers. It has been reported that exenatide and liraglutide have shown good efficacy in AD and PD. Exenatide can promote hippocampal neurogenesis, improve memory performance, and reduce the degree of tau hyperphosphorylation. Liraglutide can reduce insulin resistance and tau hyperphosphorylation, as well as improve hippocampal synaptic plasticity. All these effects are achieved through activating GLP-1R^42^. It is worth noting that the activation of mTOR and tau hyperphosphorylation are both signs of AD. Studies have reported a correlation between tau hyperphosphorylation and the activation of mTOR. Activation of mTOR leads to hyperphosphorylation of tau^43,44^. Overactivation of mTOR leads to dysfunction of autophagy, which, in turn, leads to the accumulation of Aβ, causing tau hyperphosphorylation, ultimately resulting in cognitive impairment^45^.

Our results are significant as they show that dietary salt leads to tau hyperphosphorylation, damages GLP-1R and activates mTOR/p70S6K pathway, which eventually leads to cognitive impairment in C57BL/6 mice. Our experiments found that GLP-1R agonists can promote autophagy by inhibiting mTOR/p70S6K pathway. However, we used GLP-1R agonists in vitro in this study, so further in vivo experiments are needed to confirm that the silence of GLP-1R can inhibit autophagy and lead to cognitive impairment by activating mTOR/p70S6K pathway. And there is a fact that in the in vivo model we did not measure brain sodium levels under HSD conditions, so that the observed in vivo effects of HSD may be indirect. Therefore, this study has made a qualitative demonstration of the cognitive impairment caused by high-salt diet, in future experiments, sodium levels in an in vivo model will be measured to investigate the quantitative level of cognitive dysfunction caused by a high-salt diet. In addition, our study did not fully explore the process by which tau phosphorylation leads to alterations in synaptic function. However, our research is still worthy of attention as our experiments have established that both GLP-1R and autophagy are effective therapeutic targets for cognitive impairment caused by dietary salt.

**Figure 10:**
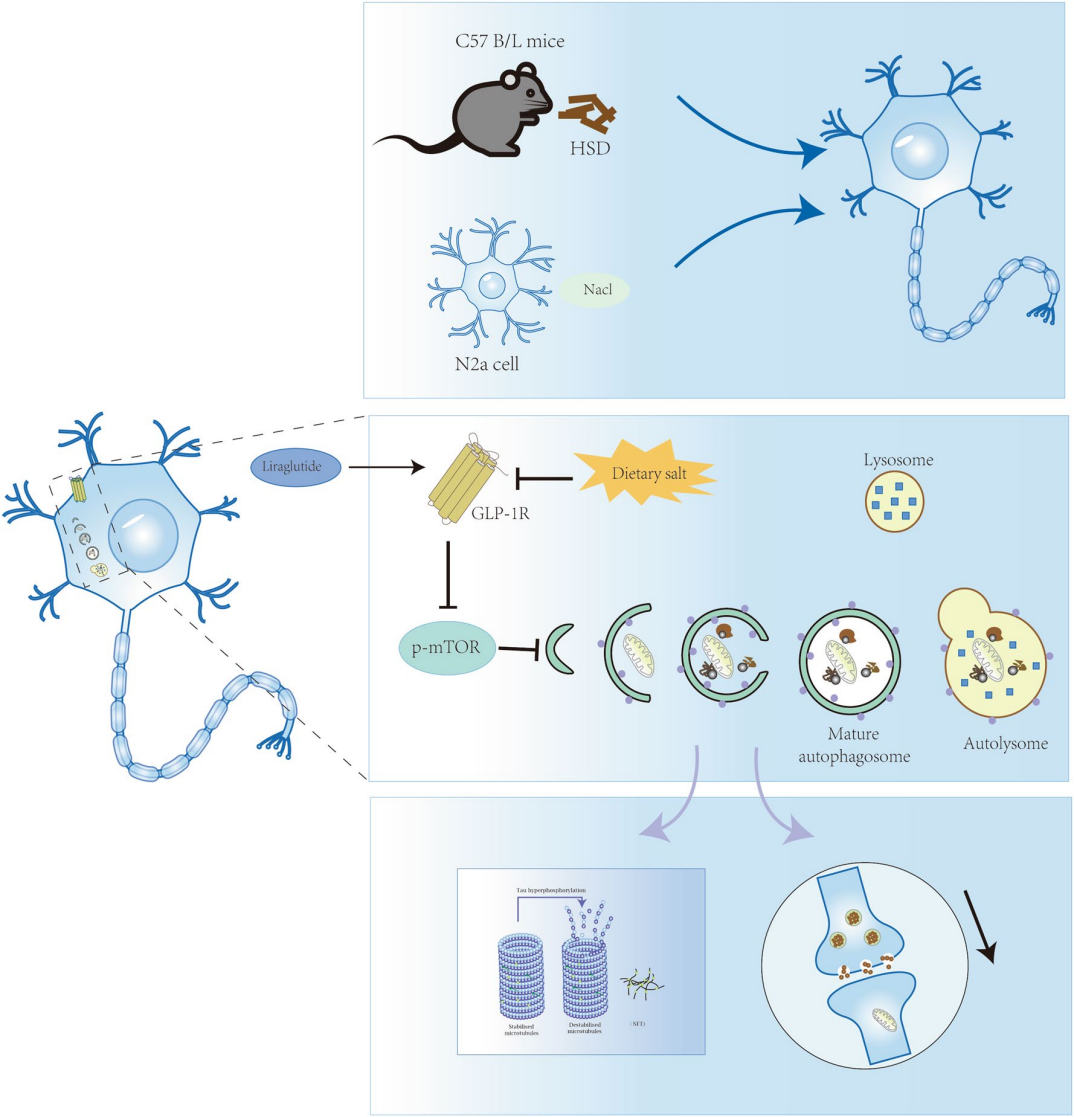
Potential mechanism of salt-induced cognition impairment. Dietary salt leads to impaired GLP-1R, which in turn leads to activation of the mTOR/p70S6K pathway, causing synapse damage and tau hyperphosphorylation, and eventually contributing to cognition impairment. HSD: High-salt diet; ND: normal diet; GLP-1R: Glucagon-like Peptide-1 receptor; mTOR: mammalian target of rapamycin; PSD95: postsynaptic density protein 95; SYP: synaptophysin; NFT: neurofibrillary tangles.

In summary, our study demonstrated that a long-term HS diet can lead to cognitive impairment and impair the level of GLP-1R, and then activate mTOR/p70S6K pathway to inhibit autophagy flux. Although the precise mechanism of tau phosphorylation and synaptic damage requires further investigation, our findings provide insights into the underlying mechanisms of cognitive dysfunction induced by a HS diet (Fig. 10) (Supplementary informations 1, 2 and 3).

### Supplementary Information

Supplementary Information 1.

Supplementary Information 2.

Supplementary Information 3.

## Data Availability

The original data that support this paper's conclusions will be provided by the corresponding author.

## Abbreviations

WB: Western blotting
GLP-1R: Glucagon-like Peptide-1 receptor
HS: High salt
HSD: High-salt diet
ND: Normal diet
mTOR: Mammalian target of rapamycin
p70S6K: Ribosomal S6 protein kinase
SYP: Synaptophysin
PSD95: Postsynaptic density 95
AD: Alzheimer's disease
PD: Parkinson's disease
MWM: Morris water maze
OFT: Open field test
SBT: Shuttle box test
PAT: Passive avoidance test
N2a: Neuro-2a
NTF: Nerve fiber tangles
