# Supplementary material for: Dietary salt promotes cognition impairment through GLP-1R/mTOR/p70S6K signaling pathway
Source: Sci Rep. 2024 Apr 4;14:7970. doi: 10.1038/s41598-024-57998-9 (PMC10995169; doi:10.1038/s41598-024-57998-9)
Supplement: Supplementary file 1 — Supplementary Information 1. [file 41598_2024_57998_MOESM1_ESM.docx]

Explanation of the original figure

In our experiments, appropriate WB bands were trimmed prior to membrane transfer according to the gel's size and the protein molecules' position, so we could not provide the whole membrane image. What we provided was the untreated western blot images of our experiment. However, there was a difference in the size of the original western blot images because our experiment involved the grouping of 2 and 3 groups, and We did not completely unify the lengths of the strips when they were cut before the experiment. Although there may be differences in the size of the bands we cropped in the original images we provided, the width of each protein was consistent due to the uniform size and thickness (1.00mm) of the gel and electrophoresis comb (10 Wells /1.00mm) used in all our WB experiments.
