# Supplementary material for: Dietary salt promotes cognition impairment through GLP-1R/mTOR/p70S6K signaling pathway
Source: Sci Rep. 2024 Apr 4;14:7970. doi: 10.1038/s41598-024-57998-9 (PMC10995169; doi:10.1038/s41598-024-57998-9)
Supplement: Supplementary file 2 — Supplementary Information 2. [file 41598_2024_57998_MOESM2_ESM.docx]

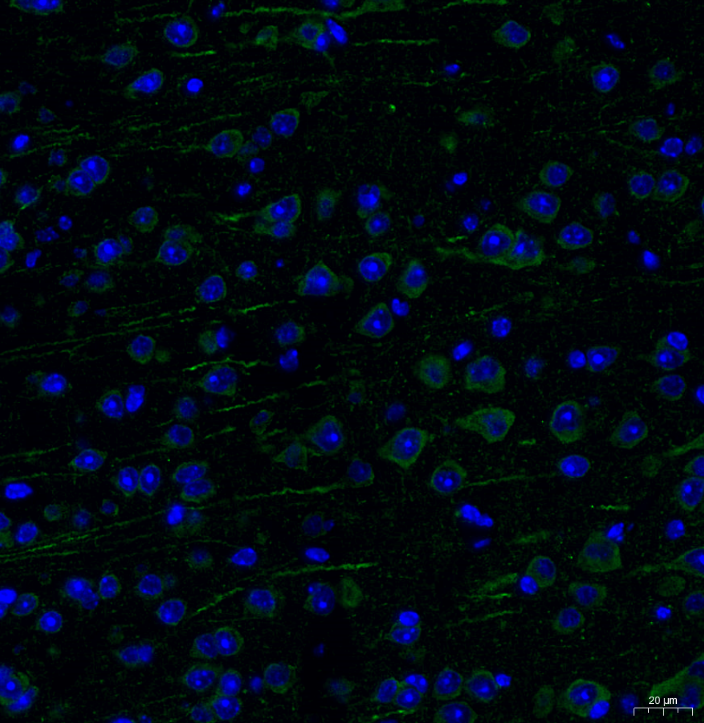


CON-GIP-1R-1-cortex


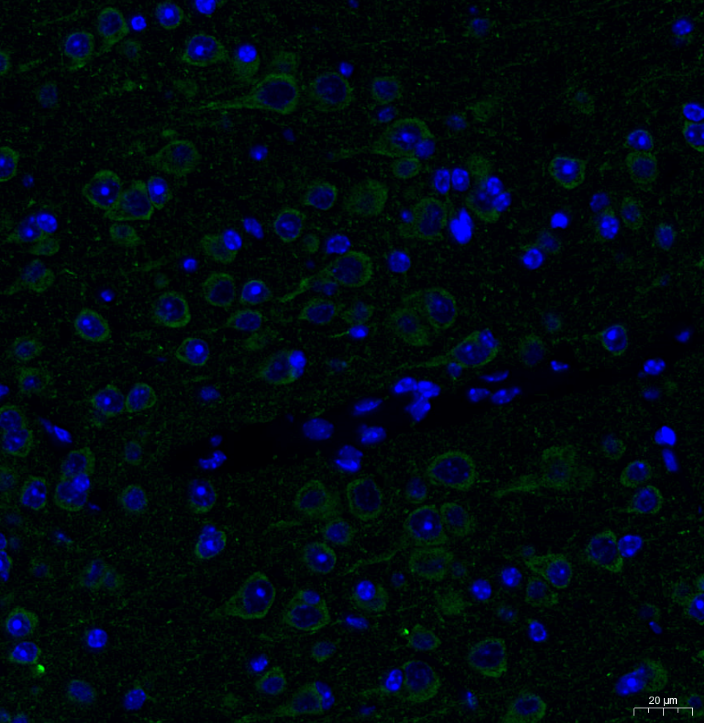


CON-GIP-1R-2-cortex


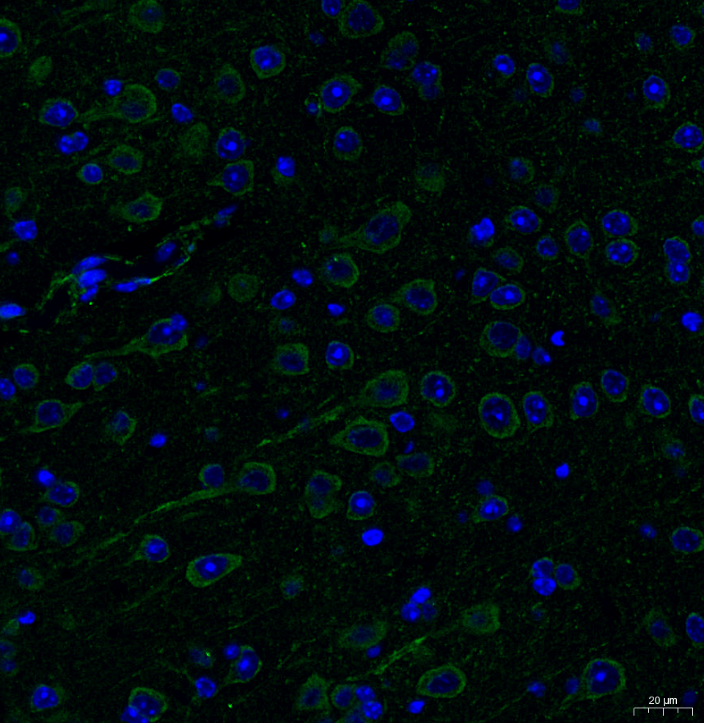


CON-GIP-1R-3-cortex


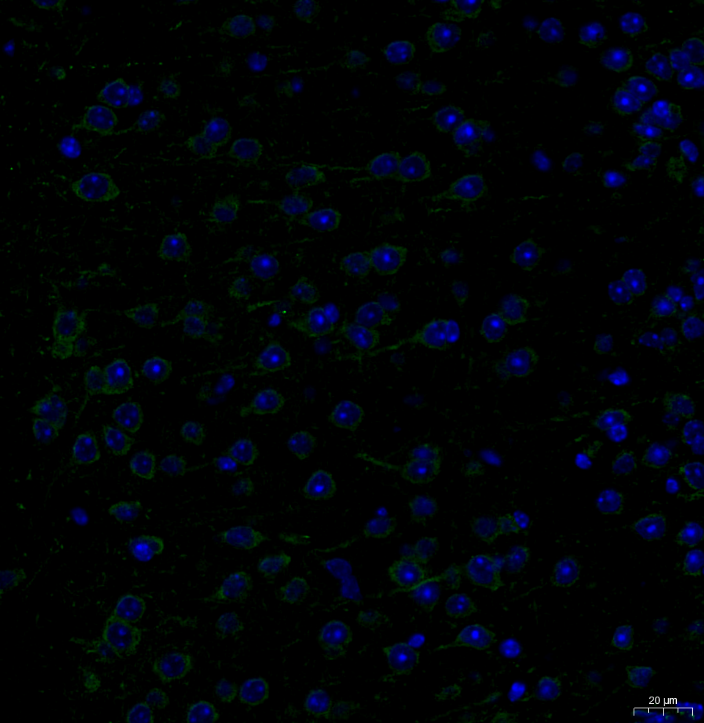


HSD-GLP-1R-1-cortex


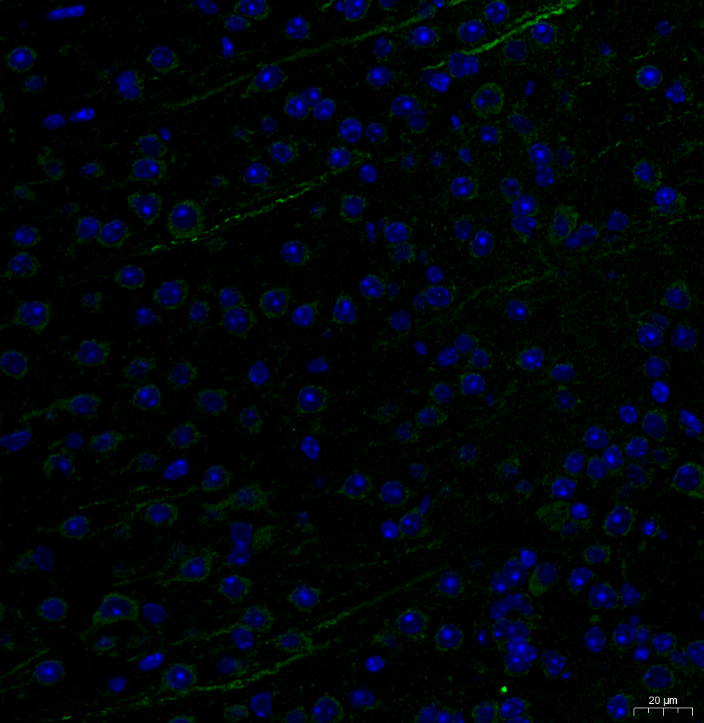


HSD-GLP-1R-2-cortex


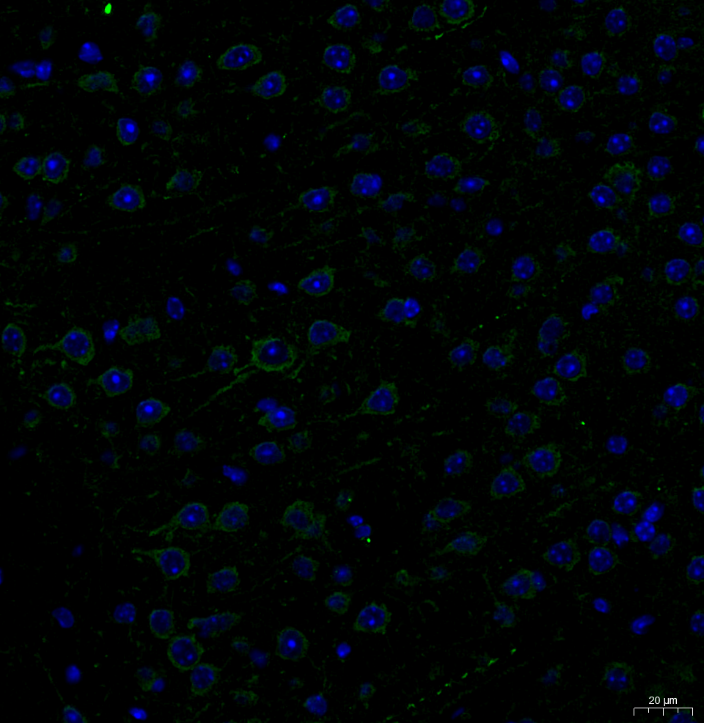


HSD-GLP-1R-3-cortex


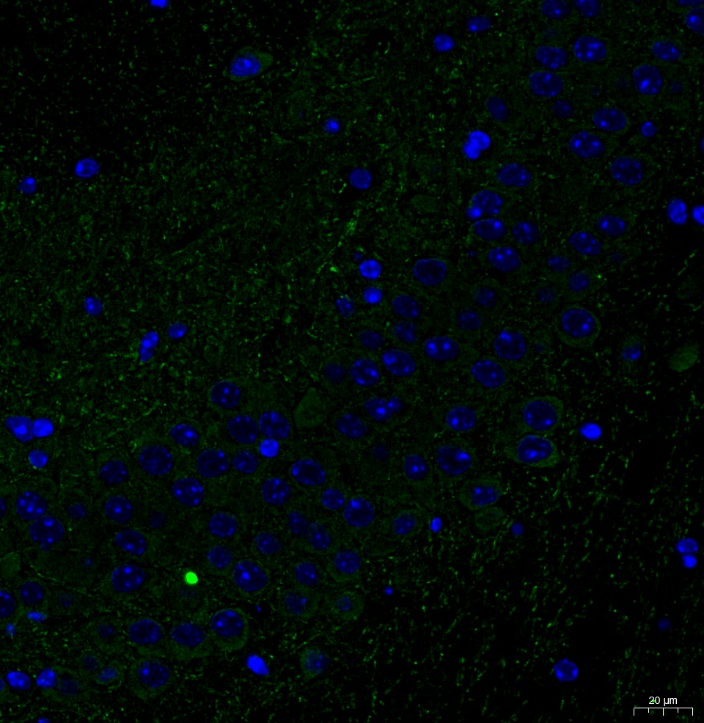


con-GLP-1R-1-HIPP


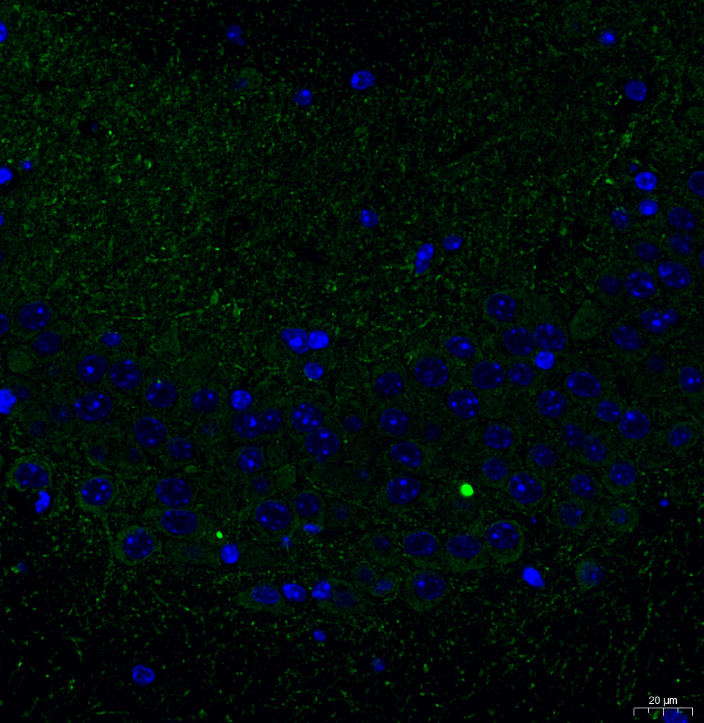


con-GLP-1R-2-HIPP


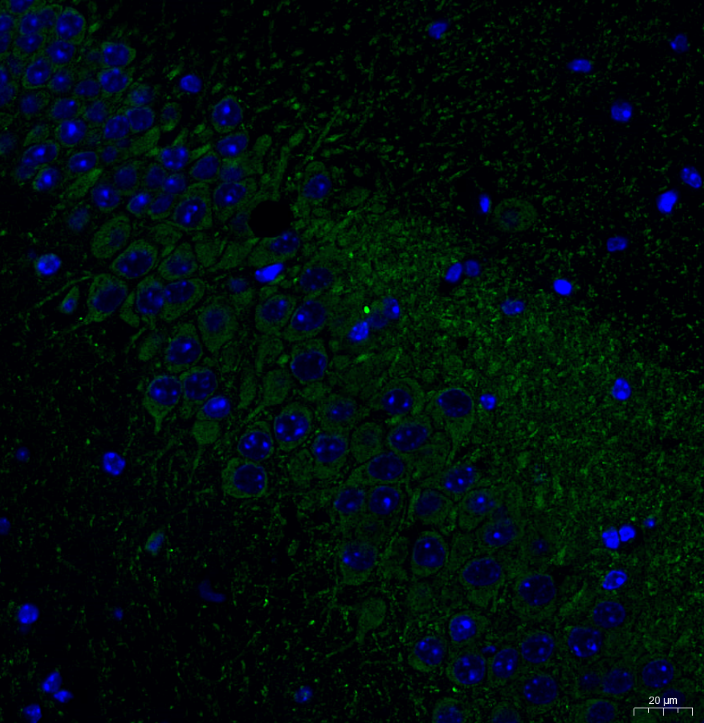


con-GLP-1R-3-HIPP


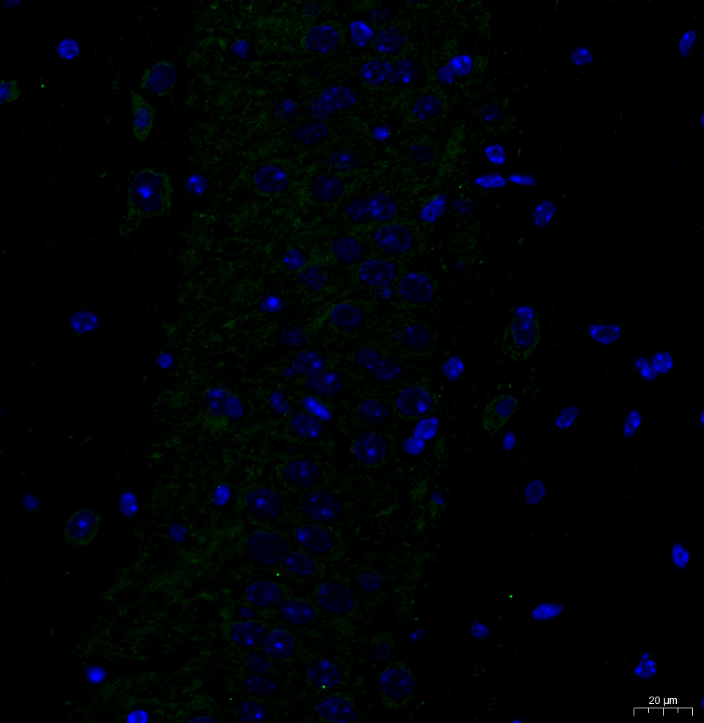


HSD-GLP-1R-1-HIPP


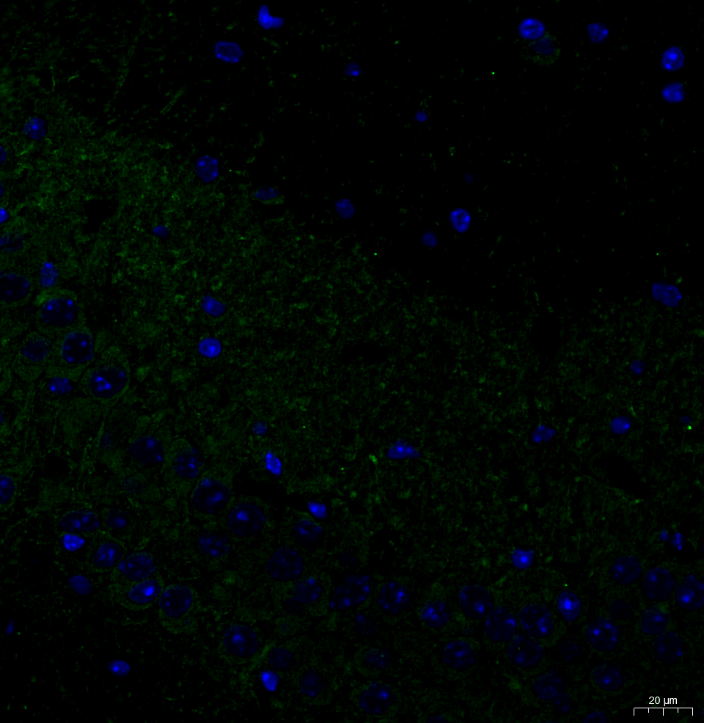


HSD-GLP-1R-2-HIPP


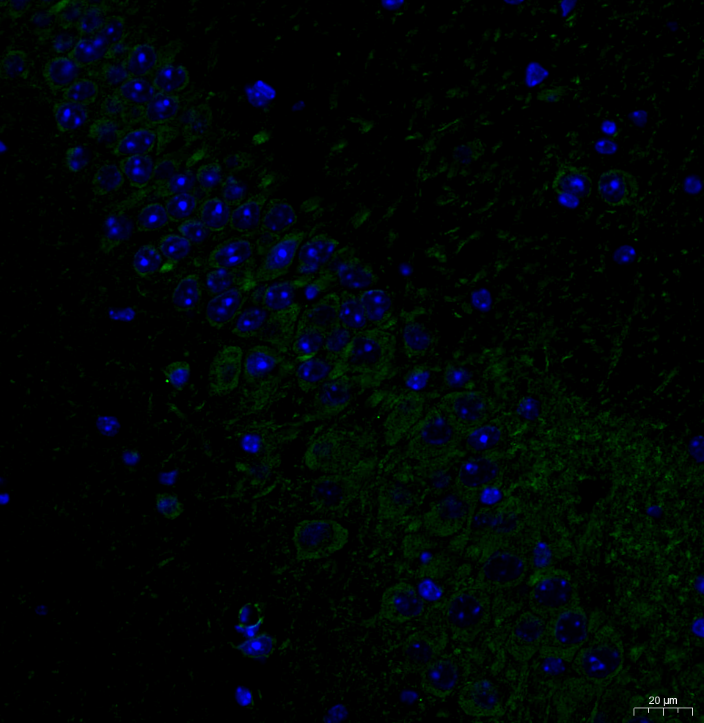


HSD-GLP-1R-3-HIPP


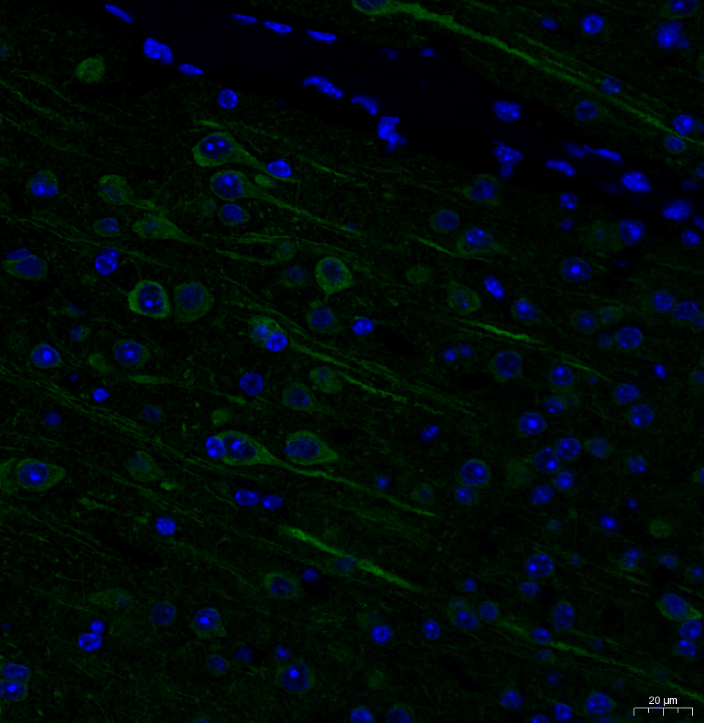


con-LC3-1-cortex


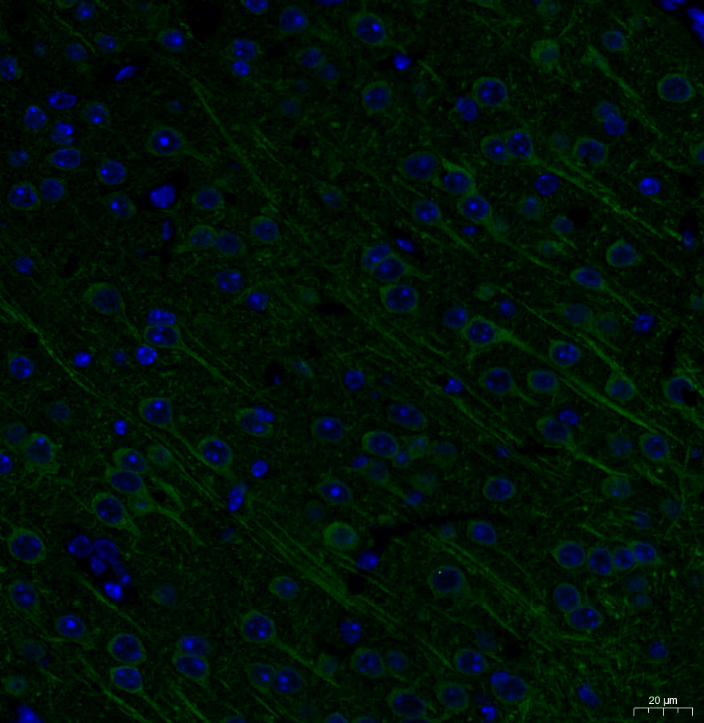


con-LC3-2-cortex


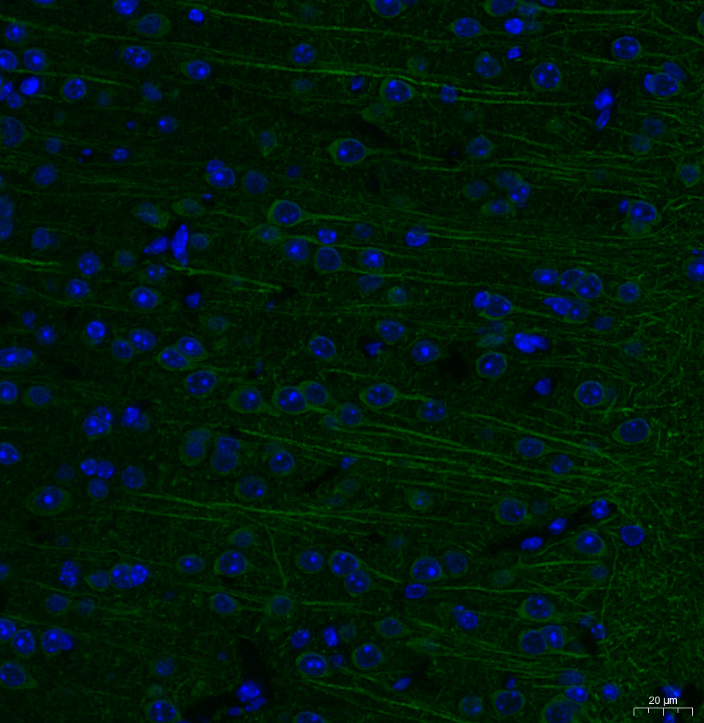


con-LC3-3-cortex


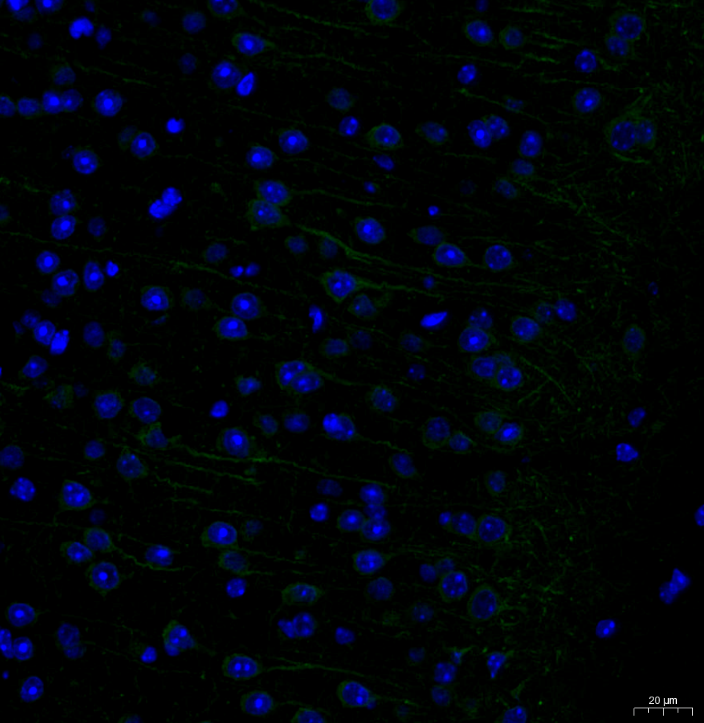


HSD-LC3-1-cortex


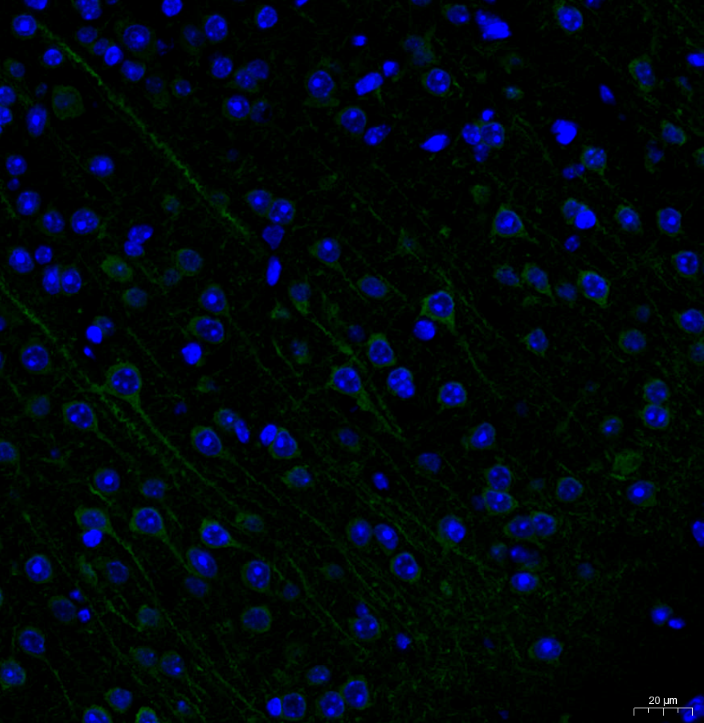


HSD-LC3-2-cortex


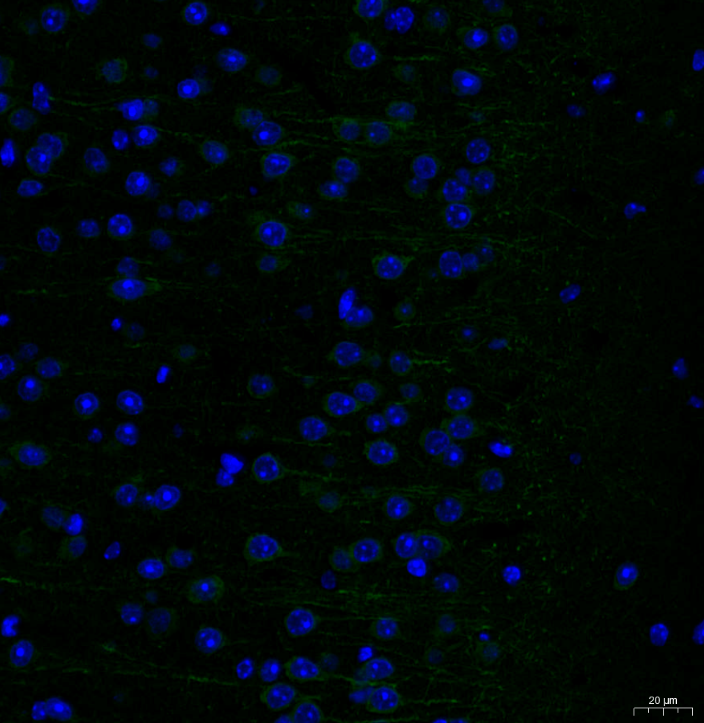


HSD-LC3-3-cortex


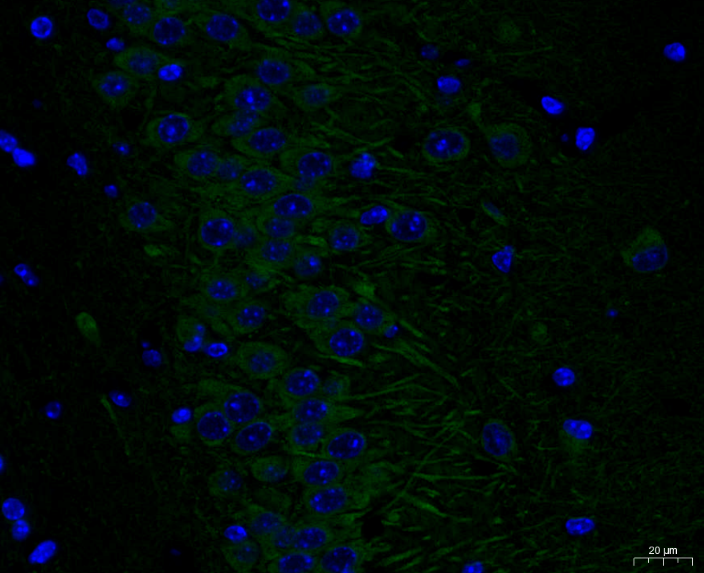


con-LC3-1-HIPP


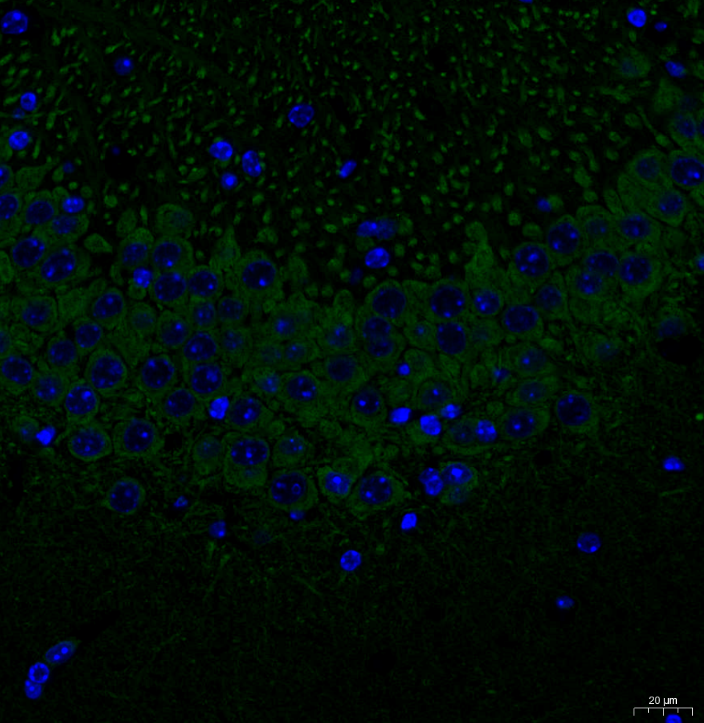


con-LC3-2-HIPP


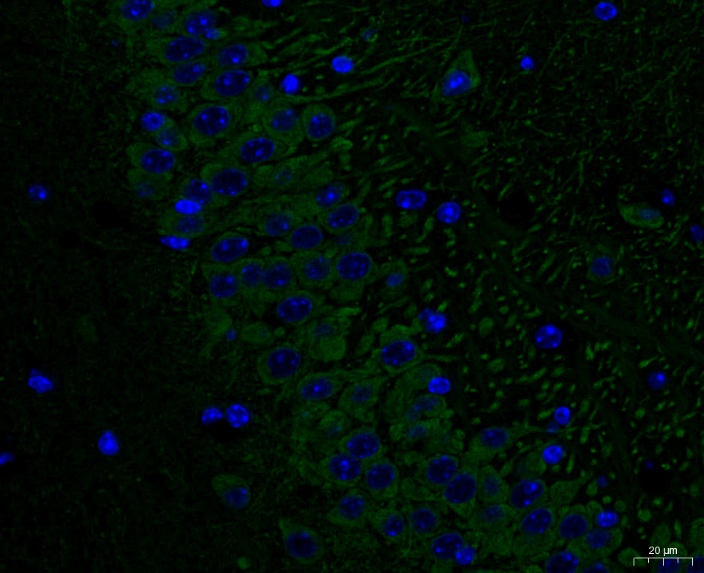


con-LC3-3-HIPP


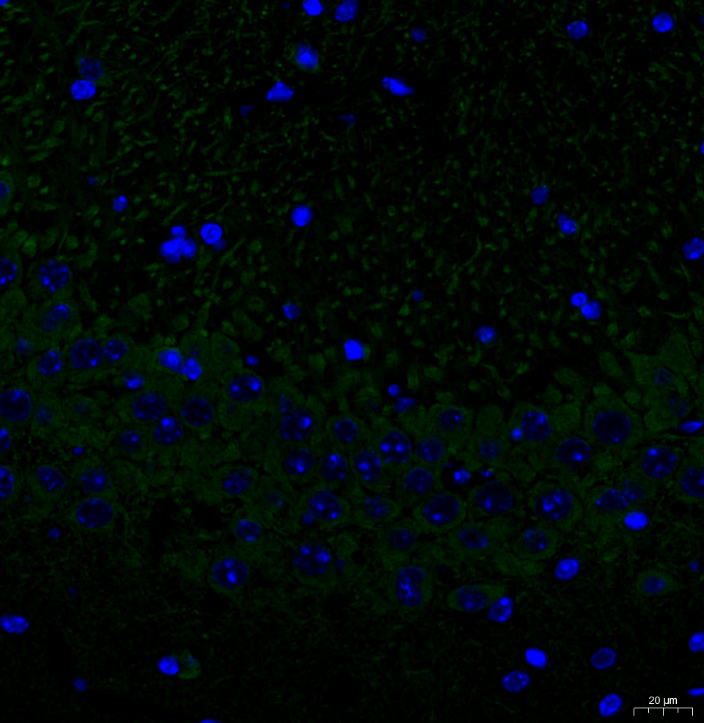


HSD-LC3-1-HIPP


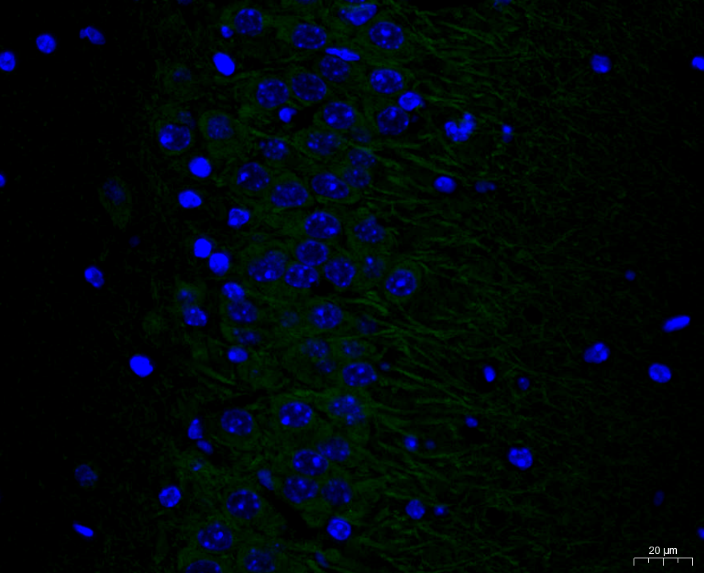


HSD-LC3-2-HIPP


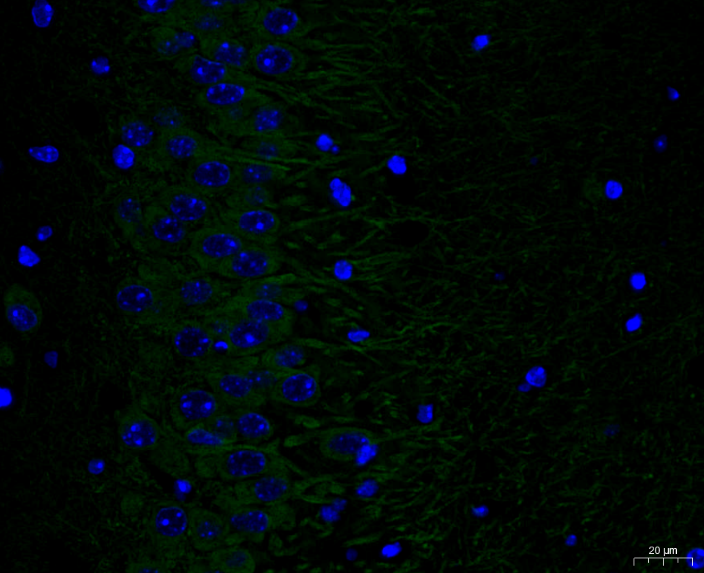


HSD-LC3-3-HIPP


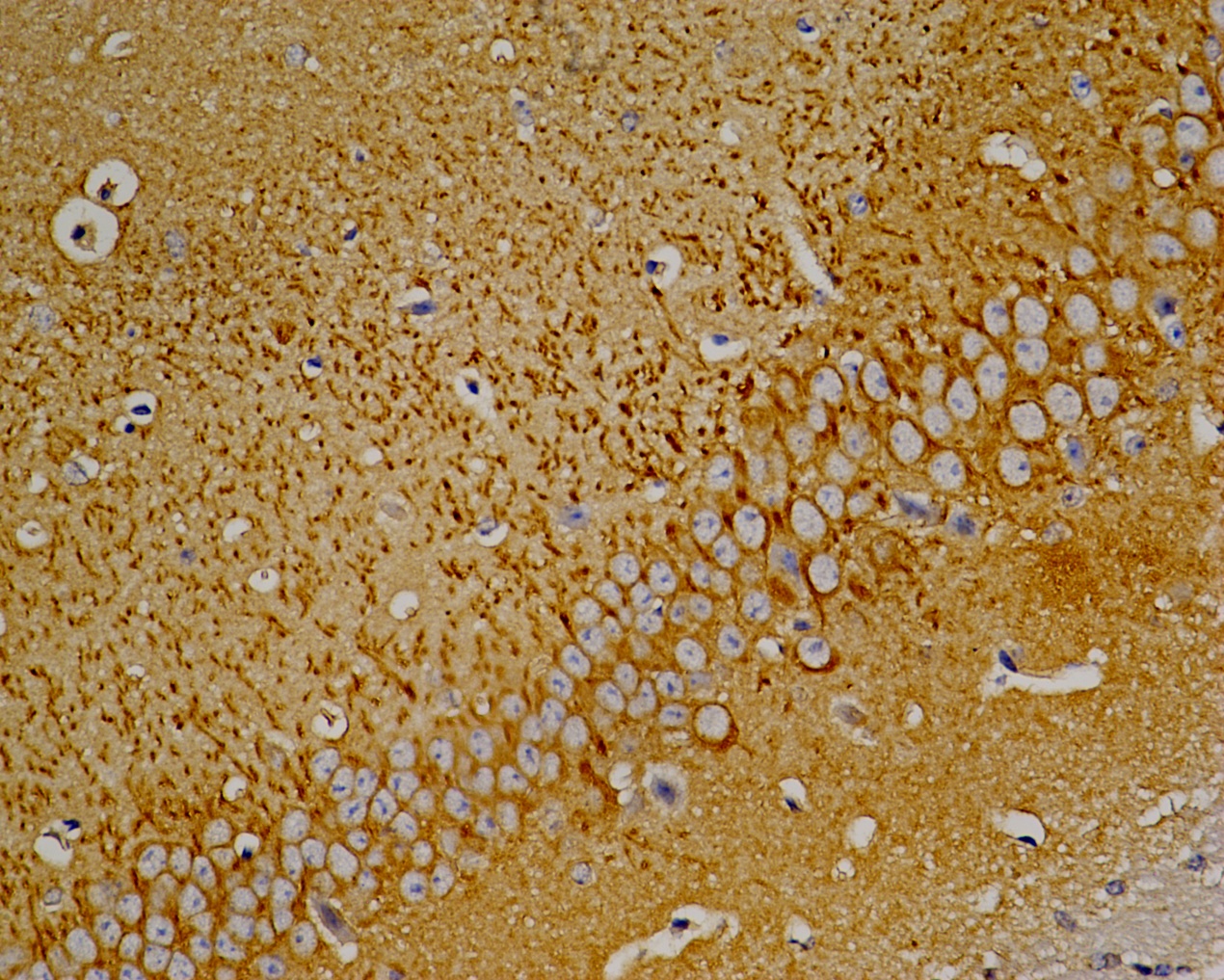


CON-GLP-1R-1


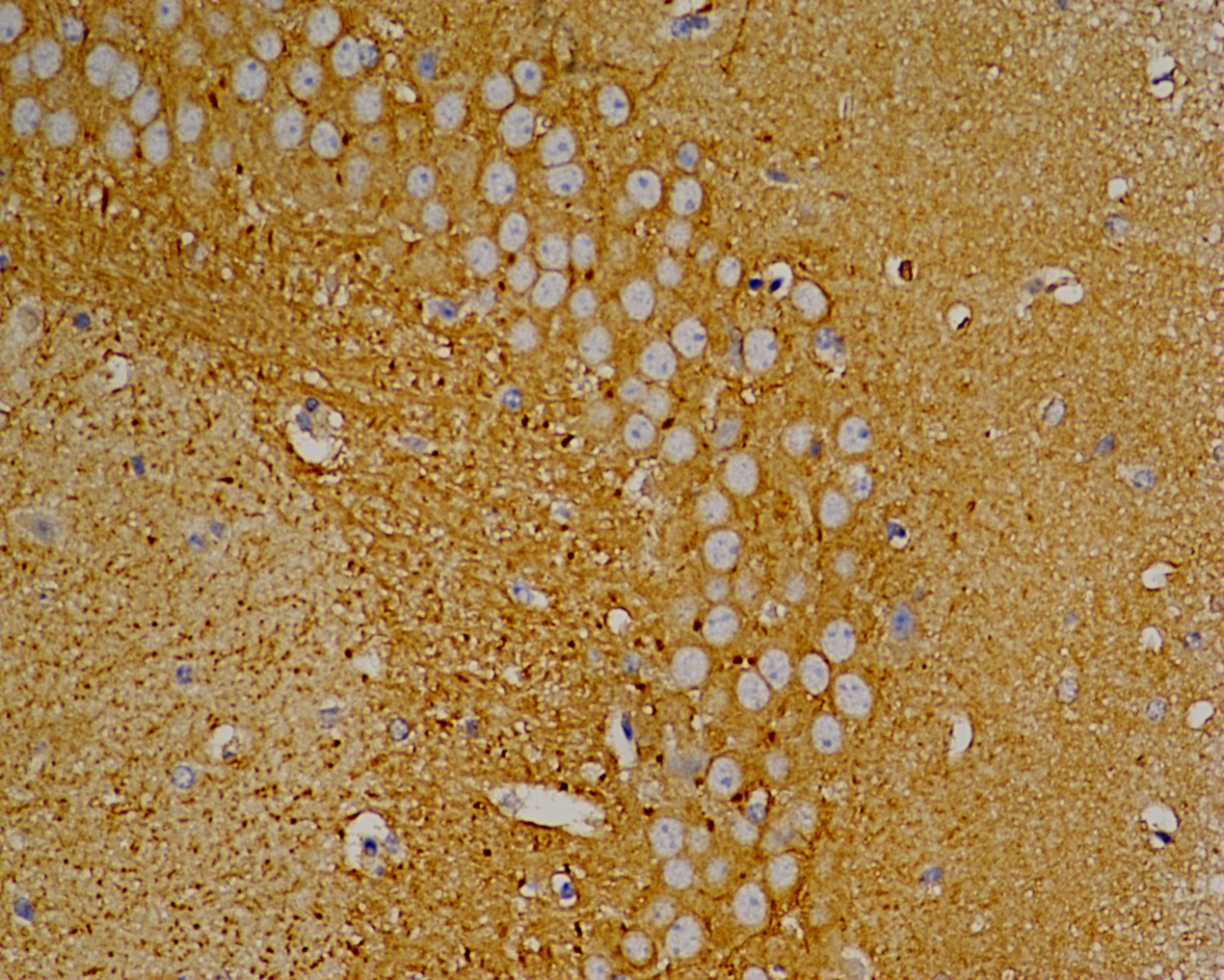


CON-GLP-1R-2


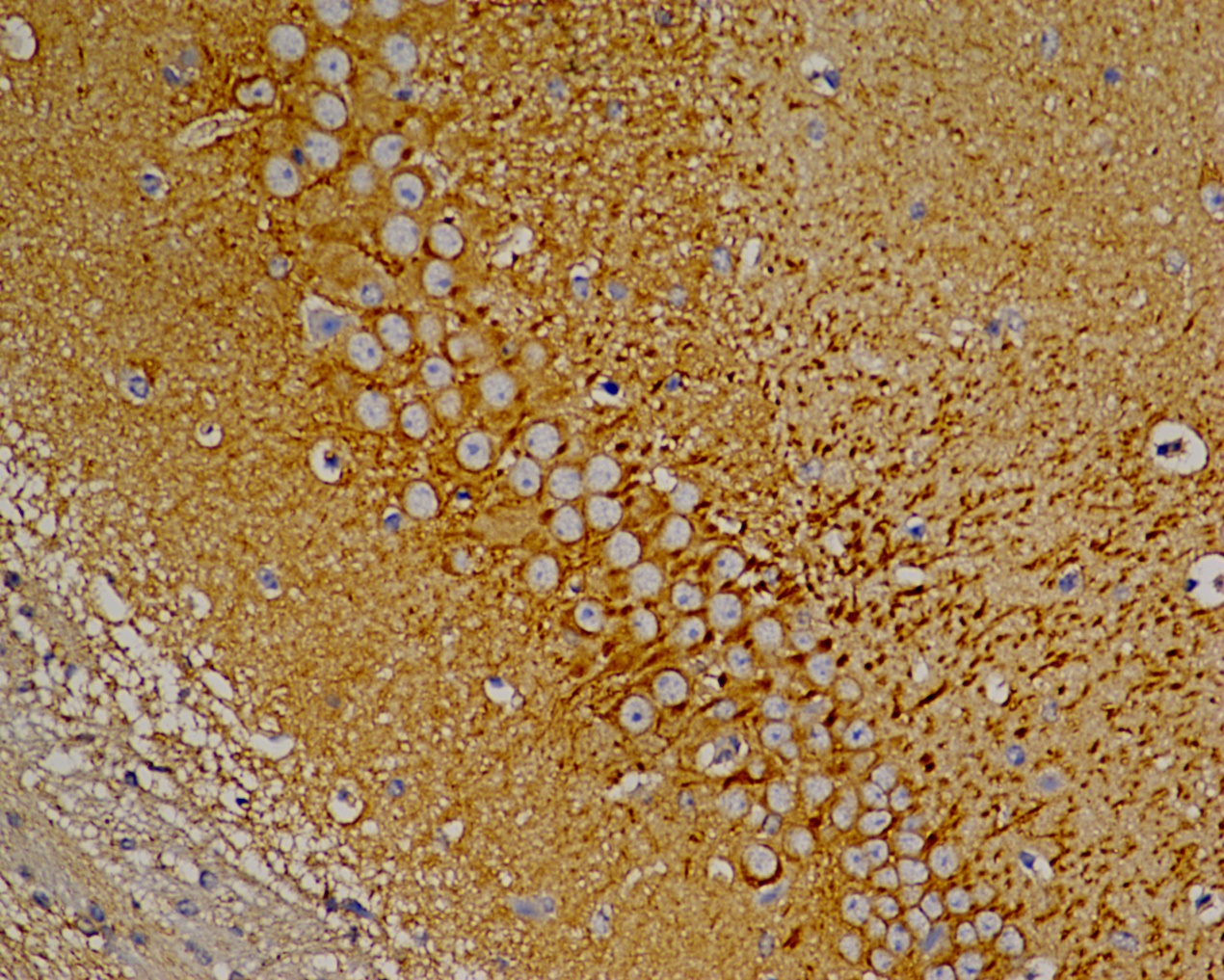


CON-GLP-1R-3


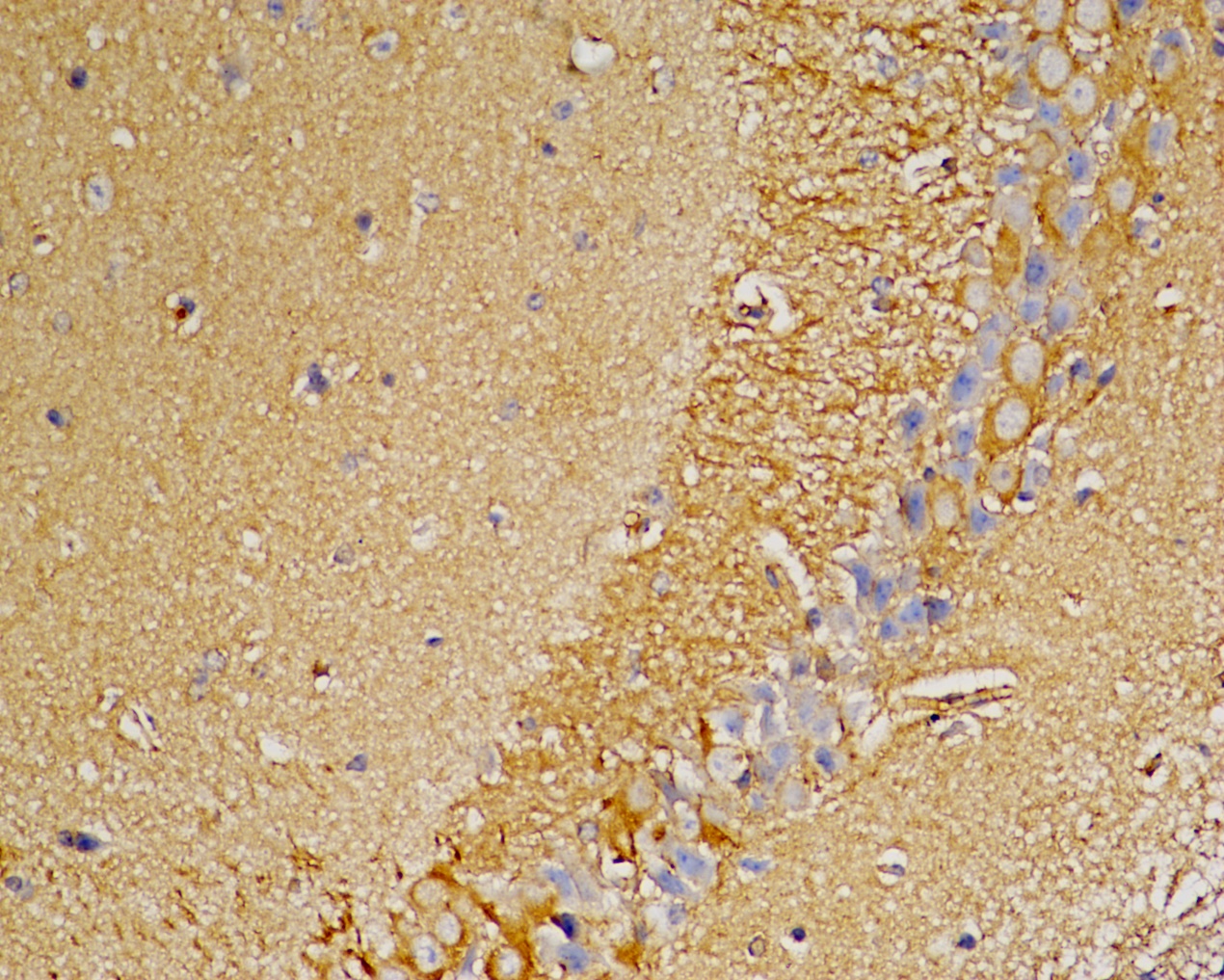


HSD-GLP-1R-1


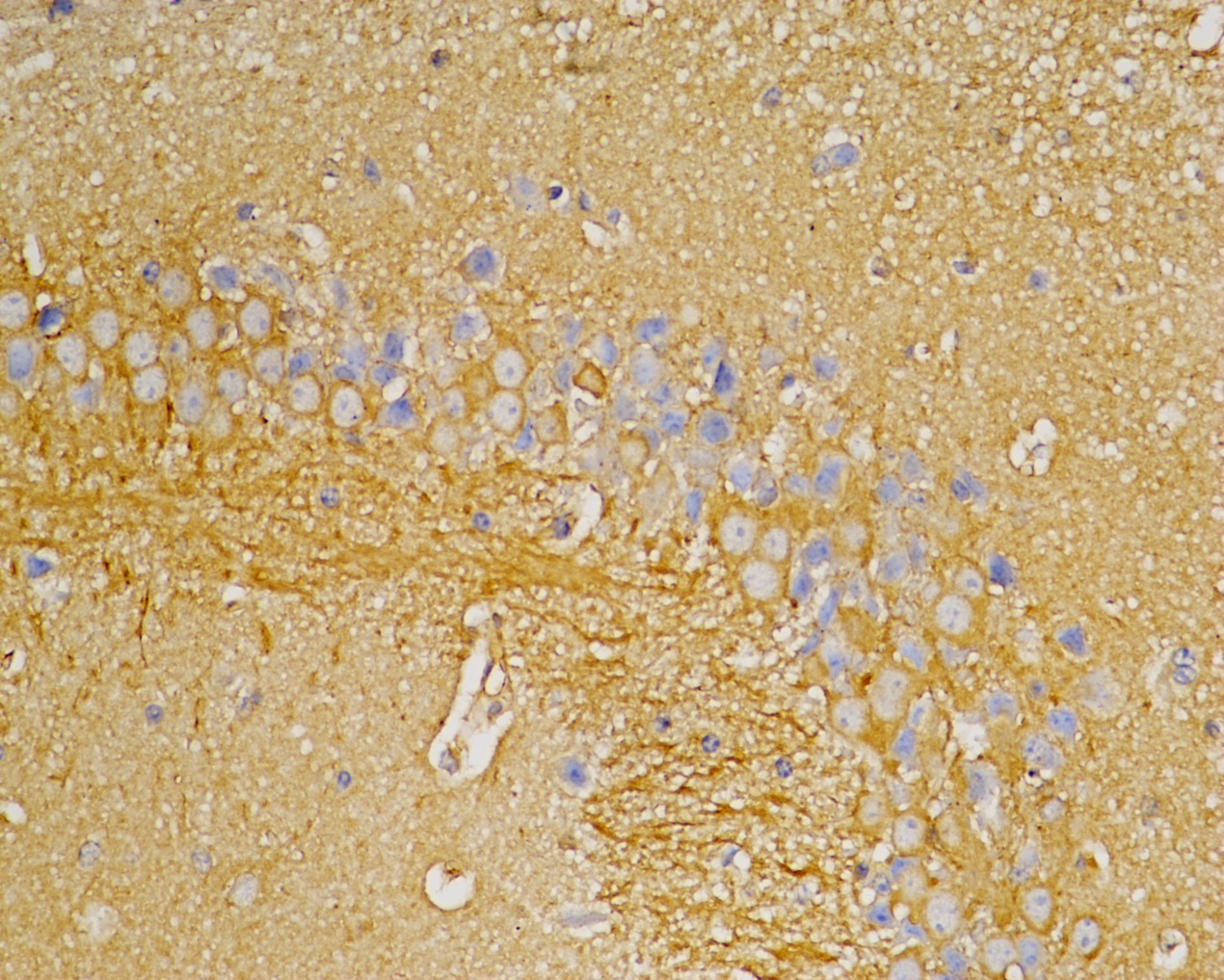


HSD-GLP-1R-2


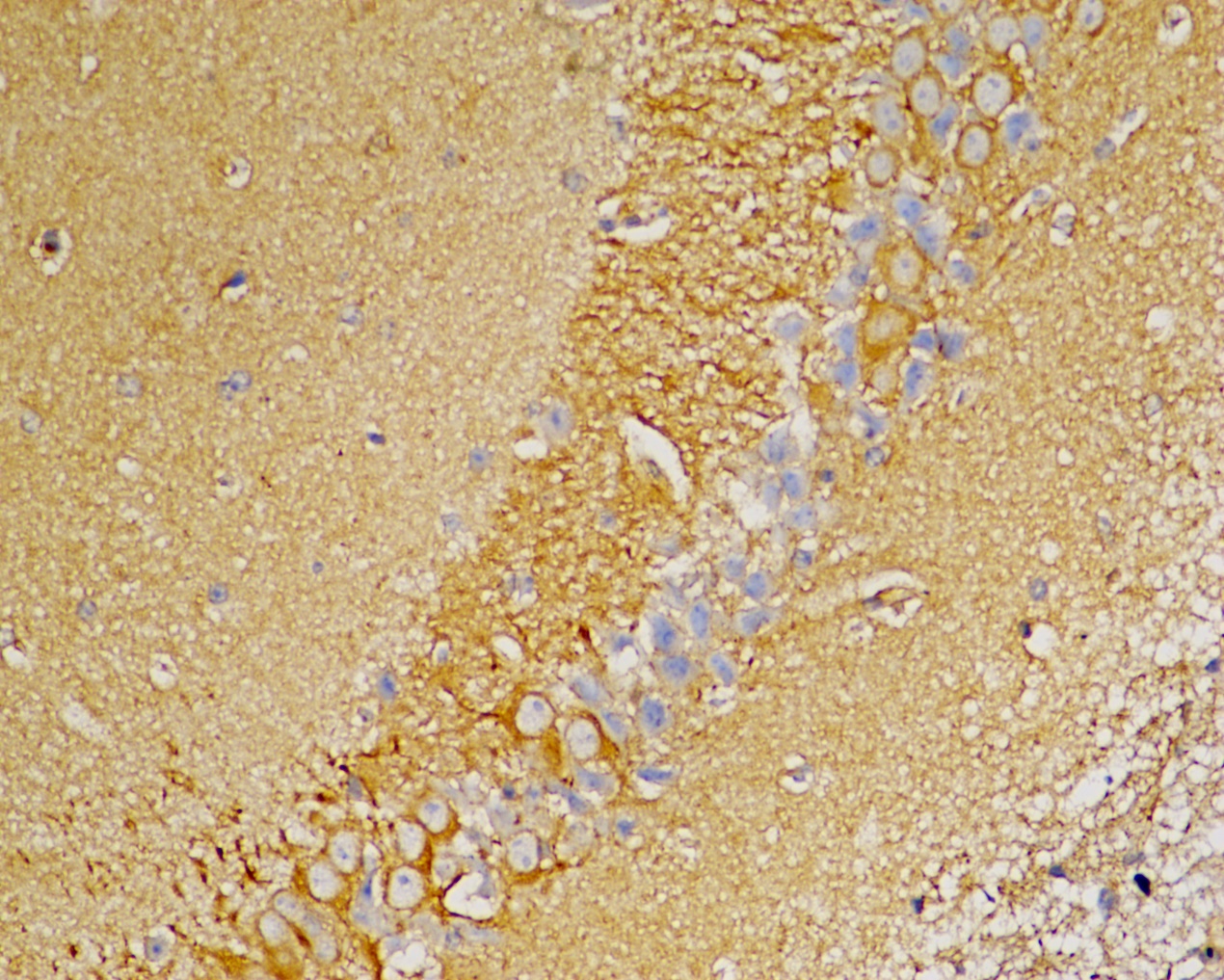


HSD-GLP-1R-3


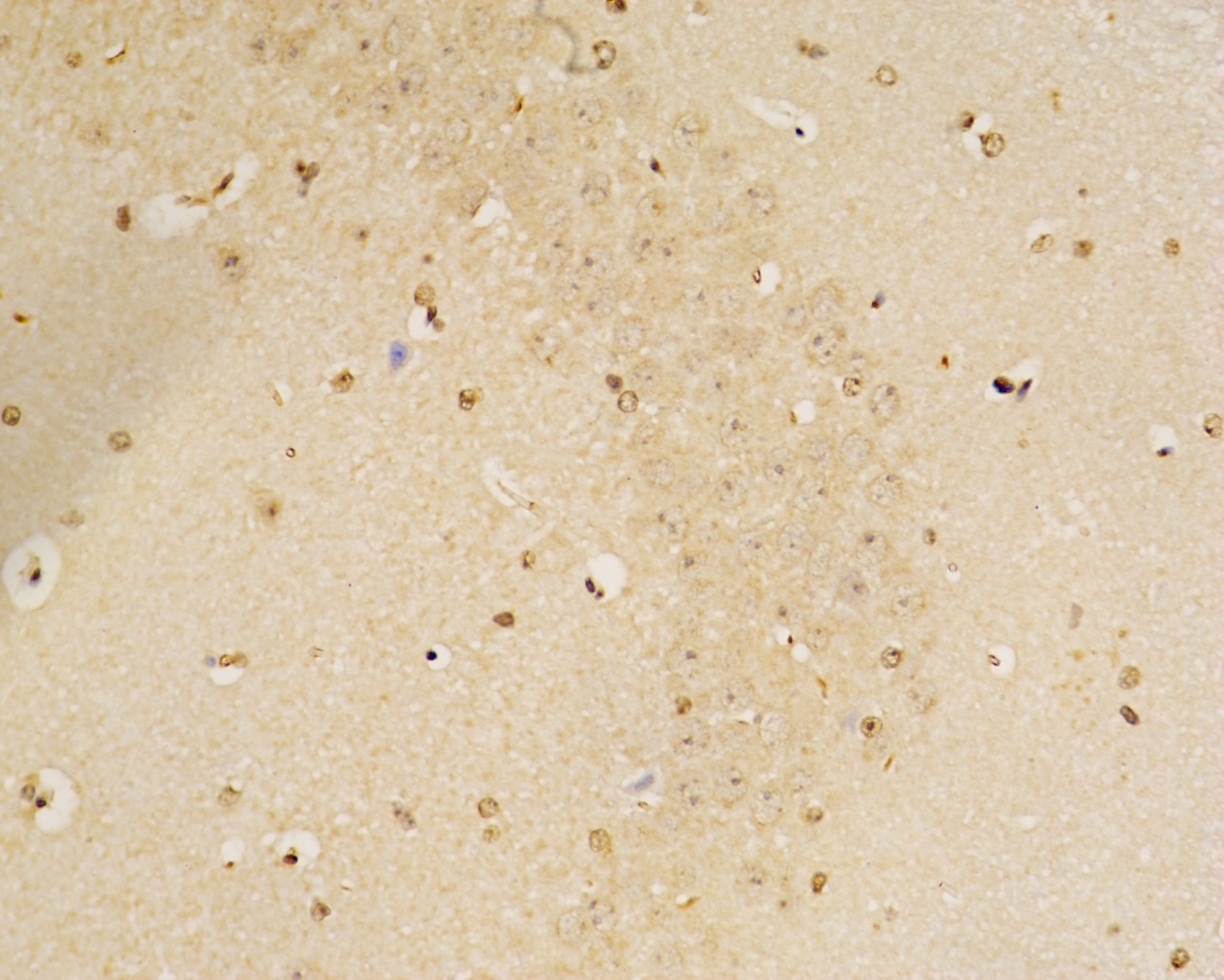


CON-LC3-1


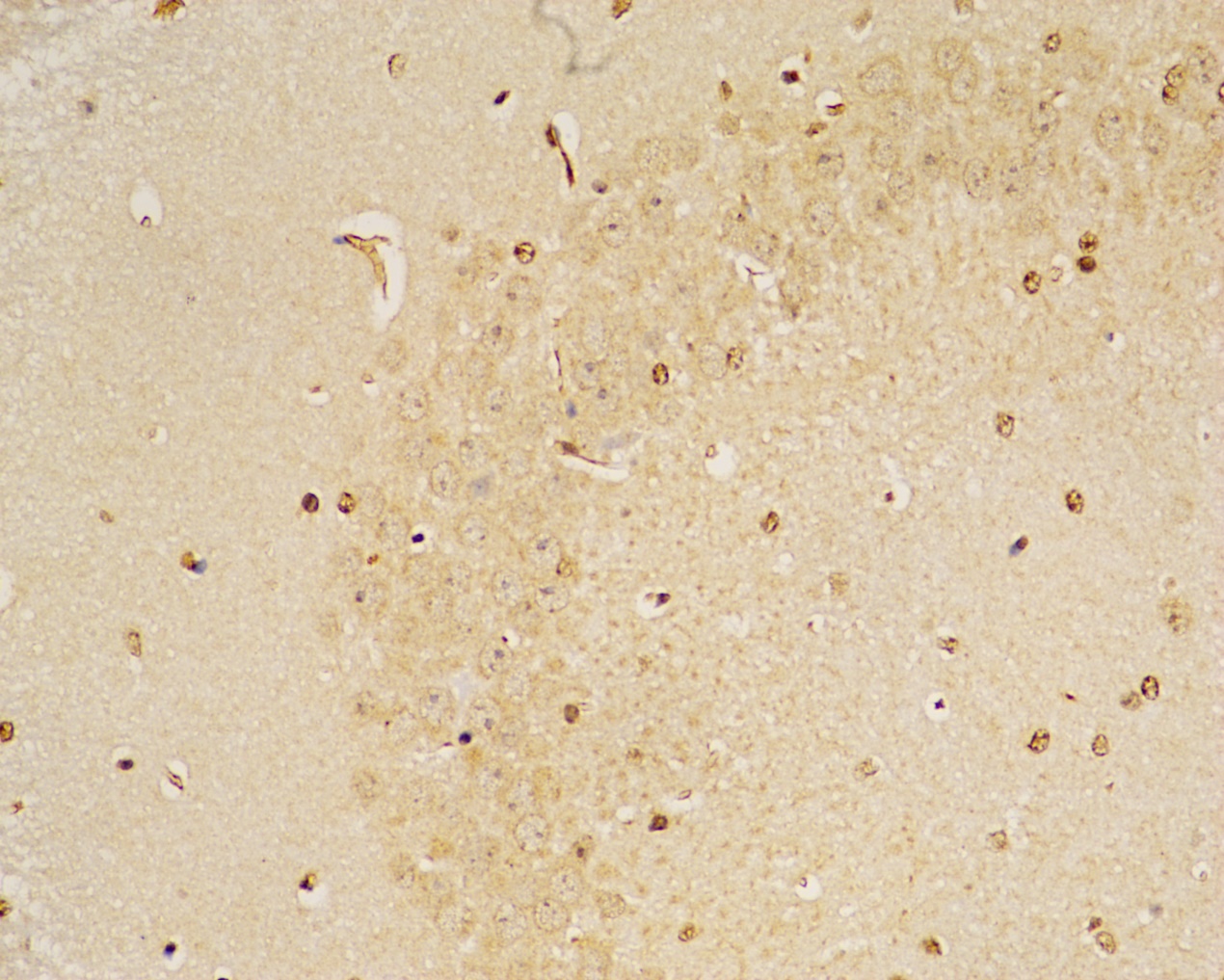


CON-LC3-2


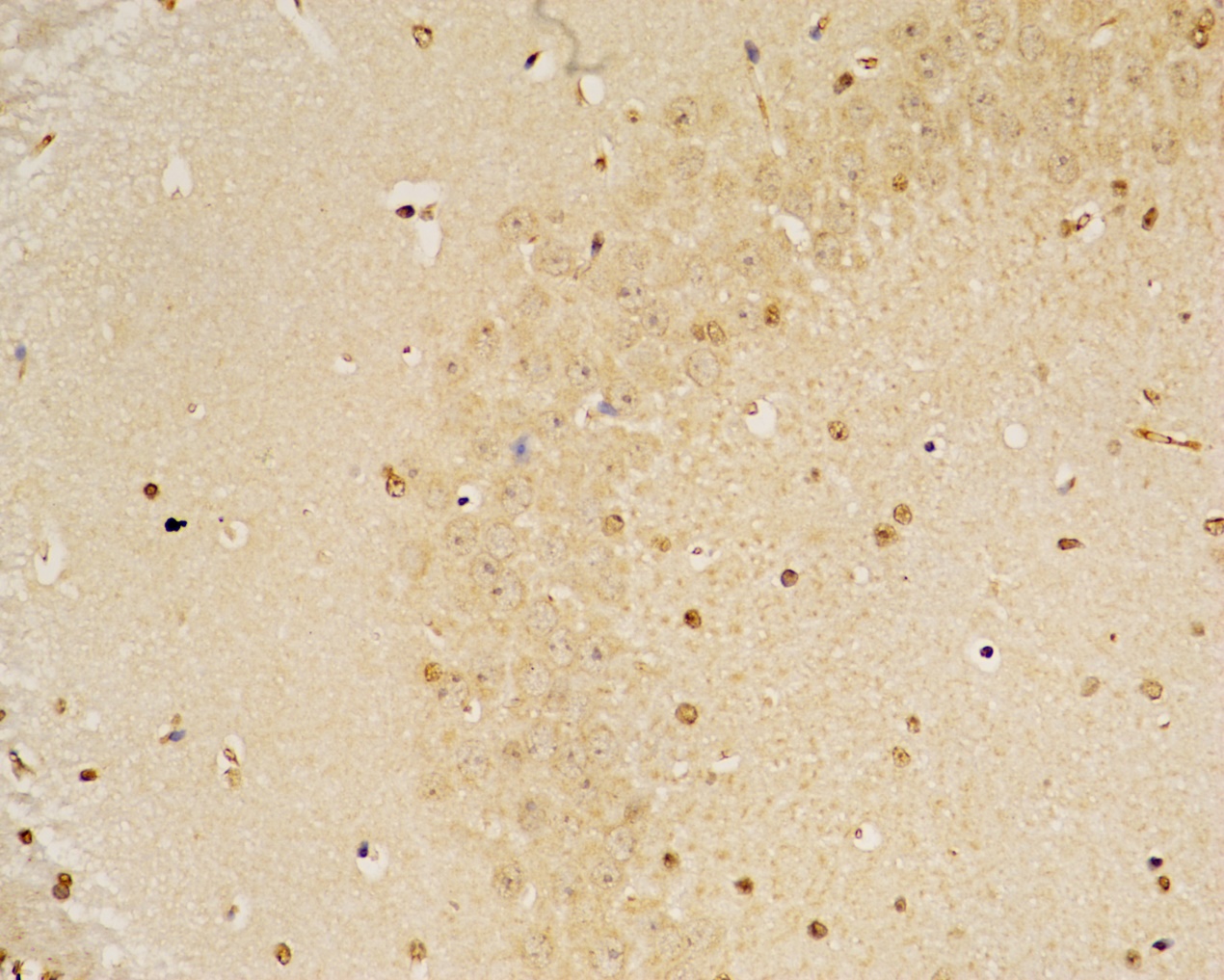


CON-LC3-3


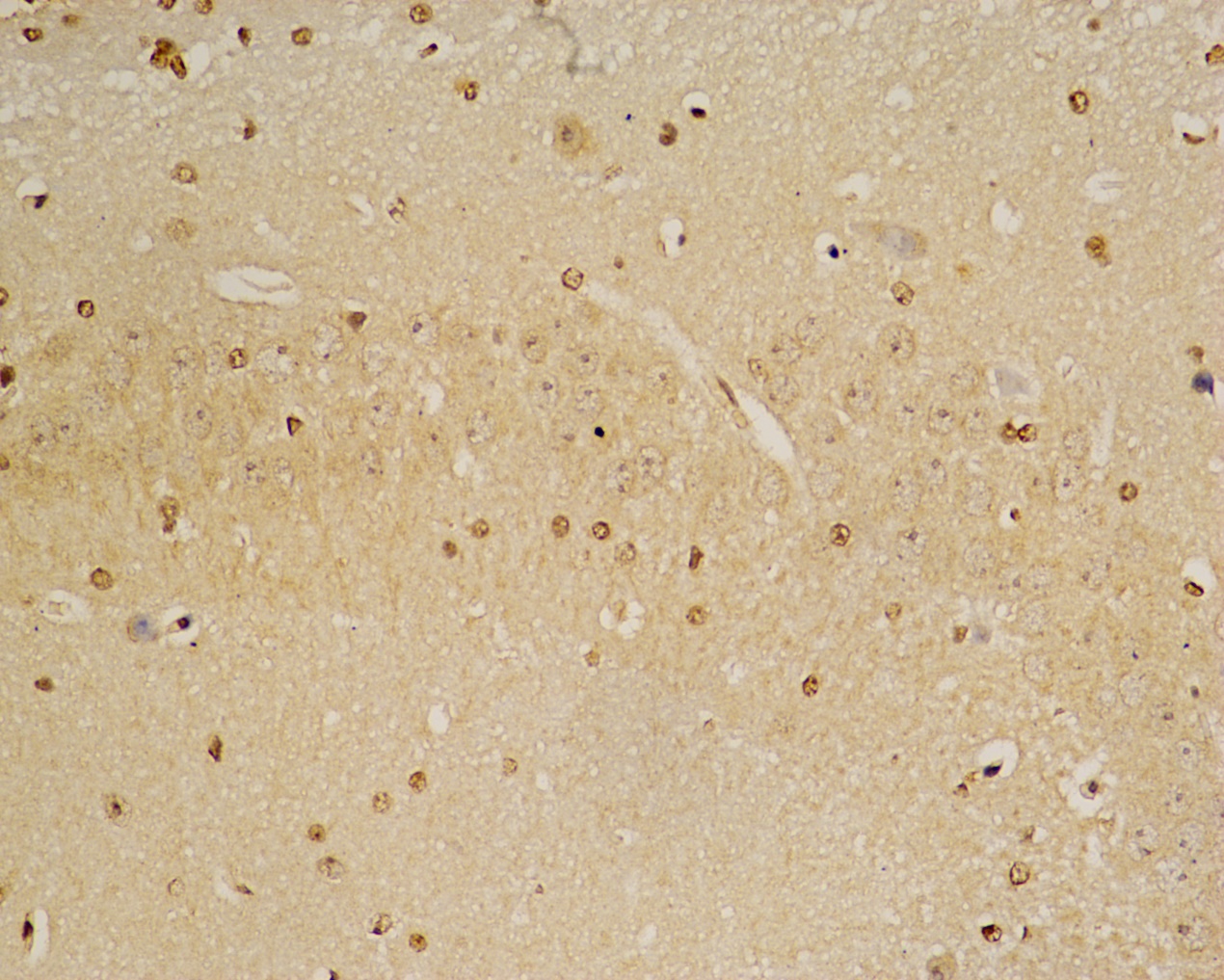


HSD-LC3-1


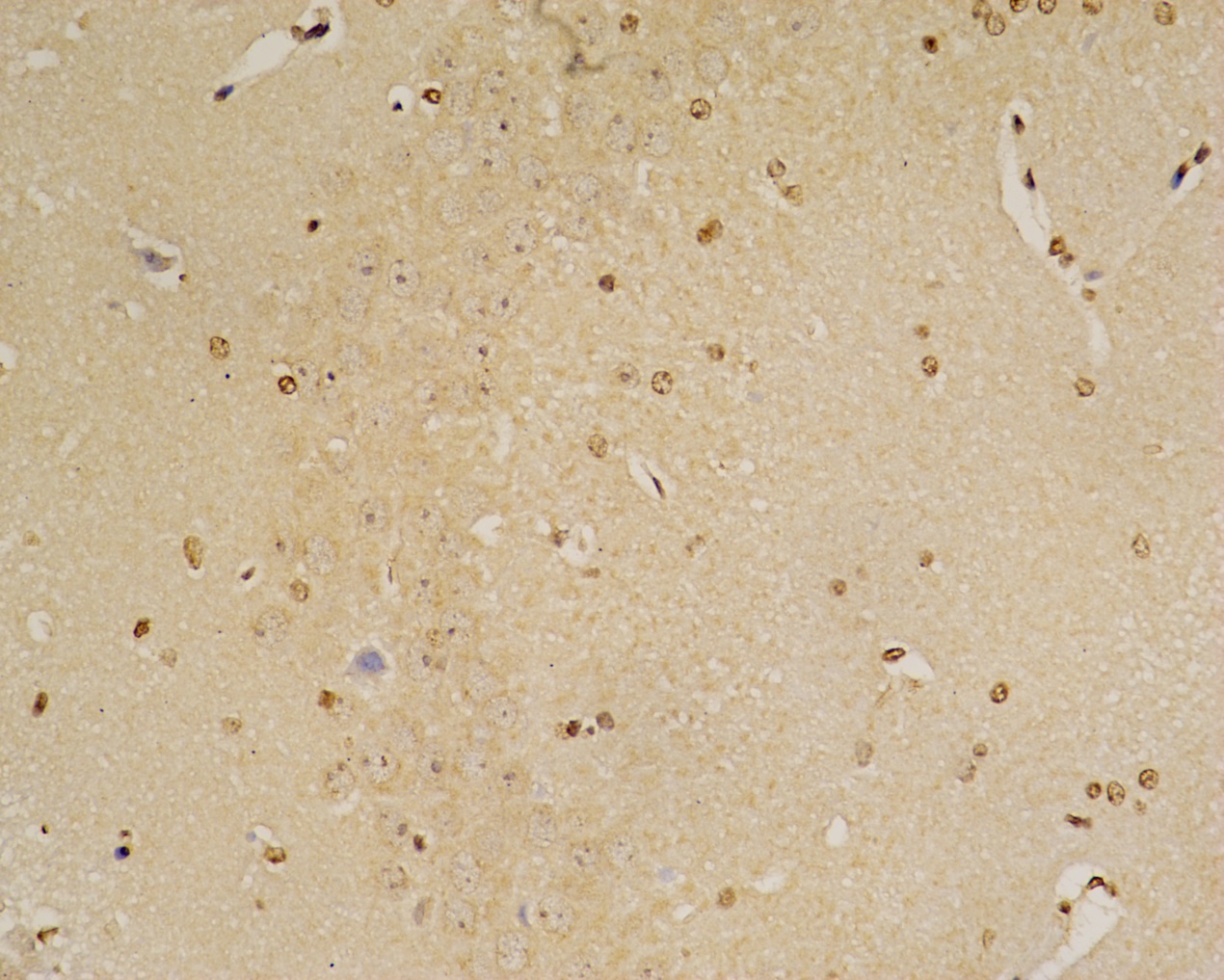


HSD-LC3-2


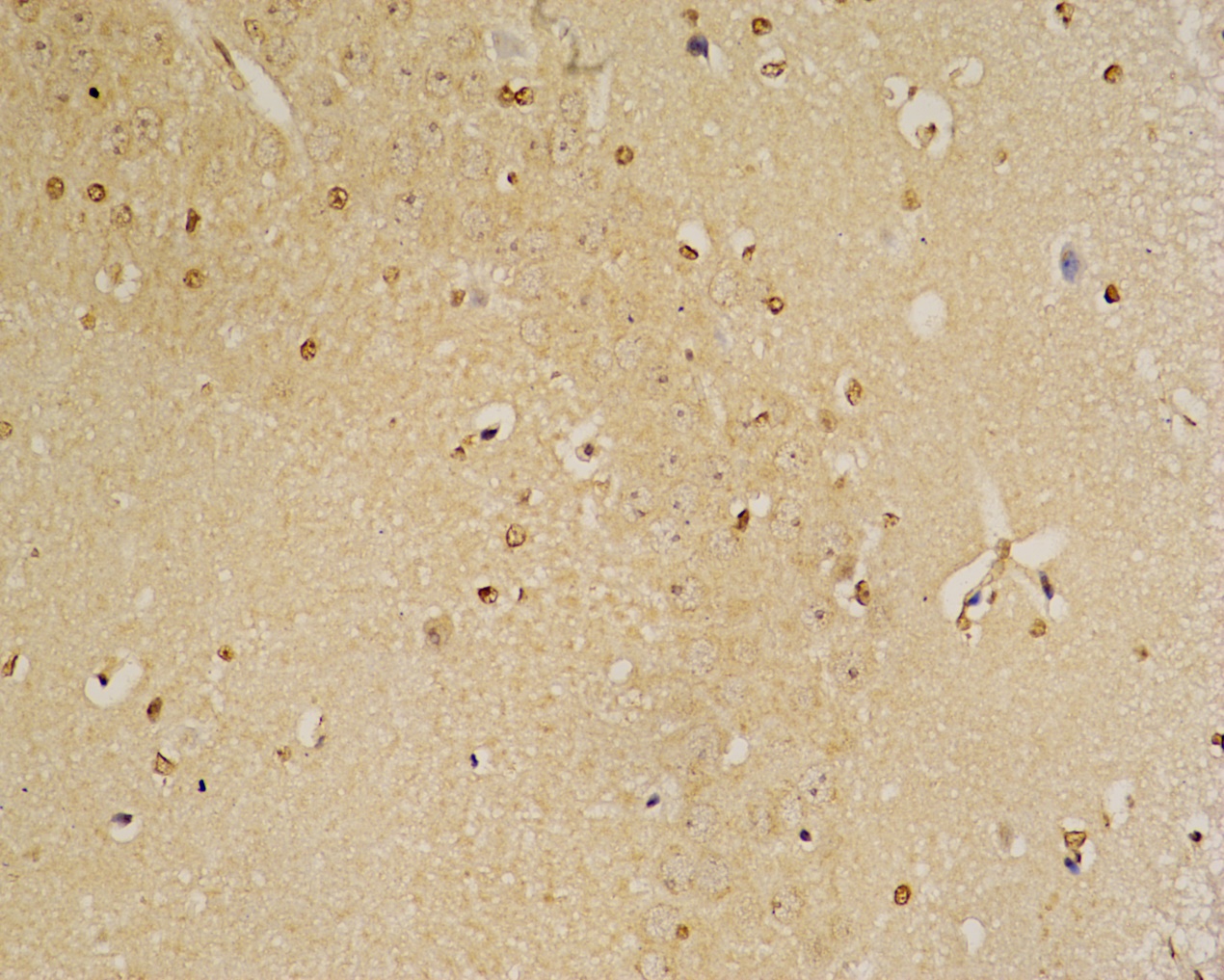


HSD-LC3-3
