# Supplementary material for: Dietary salt promotes cognition impairment through GLP-1R/mTOR/p70S6K signaling pathway
Source: Sci Rep. 2024 Apr 4;14:7970. doi: 10.1038/s41598-024-57998-9 (PMC10995169; doi:10.1038/s41598-024-57998-9)
Supplement: Supplementary file 3 — Supplementary Information 3. [file 41598_2024_57998_MOESM3_ESM.docx]

Fig4a-Beclin1-1





Fig4a-Beclin1-2





Fig4a-Beclin1-3





Fig4a-GLP-1R-1





Fig4a-GLP-1R-2





Fig4a-GLP-1R-3





Fig4a-LC3-1





Fig4a-LC3-2





Fig4a-LC3-3





Fig4a-mTOR-1





Fig4a-mTOR-2





Fig4a-mTOR-3





Fig4a-P62-1





Fig4a-P62-2





Fig4a-P62-3





Fig4a-P70S6K-1





Fig4a-P70S6K-2





Fig4a-P70S6K-3





Fig4a-P-mTOR-1





Fig4a-P-mTOR-2





Fig4a-P-mTOR-3





Fig4a-P-P70S6K-1





Fig4a-P-P70S6K-1





Fig4a-P-P70S6K-1





Fig4b-Beclin1-1





Fig4b-Beclin1-2





Fig4b-Beclin1-3





Fig4b-GLP-1R-1





Fig4b-GLP-1R-2





Fig4b-GLP-1R-3





Fig4b-LC3-1





Fig4b-LC3-2


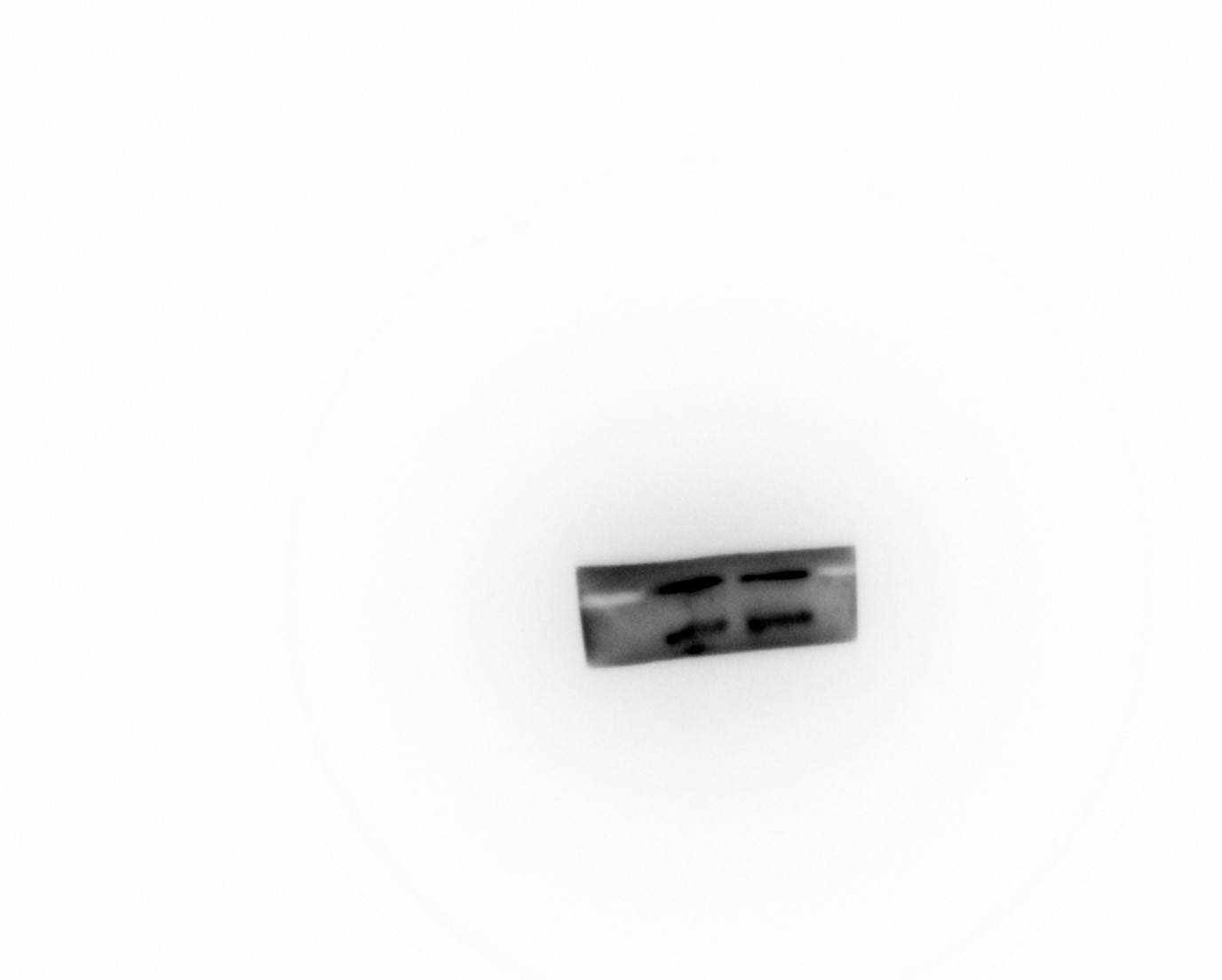


Fig4b-LC3-3





Fig4b-mTOR-1





Fig4b-mTOR-2





Fig4b-mTOR-3





Fig-4b-P62-1





Fig-4b-P62-2


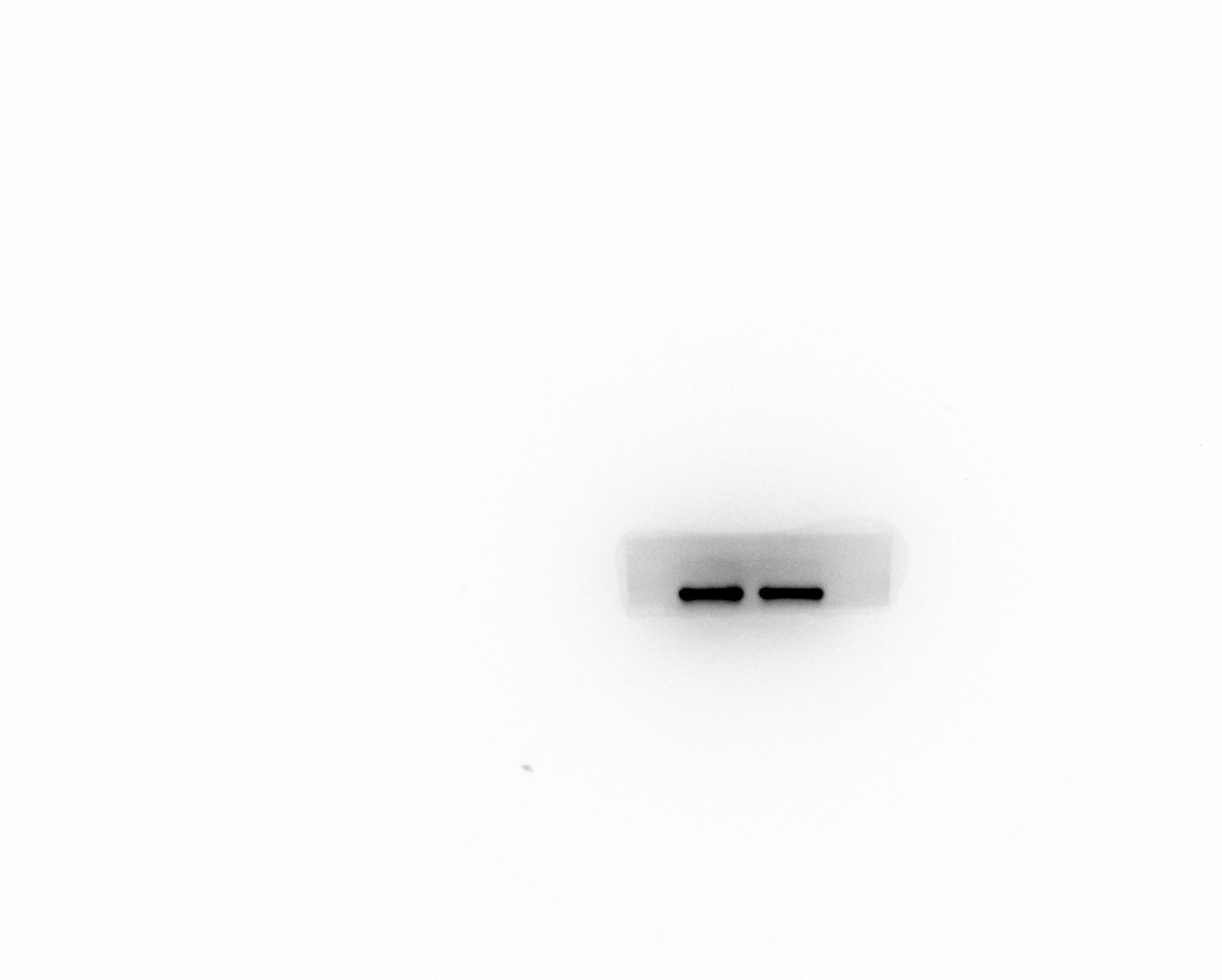


Fig-4b-P62-3





Fig4b-P70S6K-1





Fig4b-P70S6K-2





Fig4b-P70S6K-3





Fig4b-P-mTOR-1





Fig4b-P-mTOR-2





Fig4b-P-mTOR-3





Fig4b-P-P70S6K-1





Fig4b-P-P70S6K-2





Fig4b-P-P70S6K-3





Fig7-Beclin1-1





Fig7-Beclin1-2





Fig7-Beclin1-3





Fig7-GLP-1R-1





Fig7-GLP-1R-2





Fig7-GLP-1R-3





Fig7-LC3-1





Fig7-LC3-2


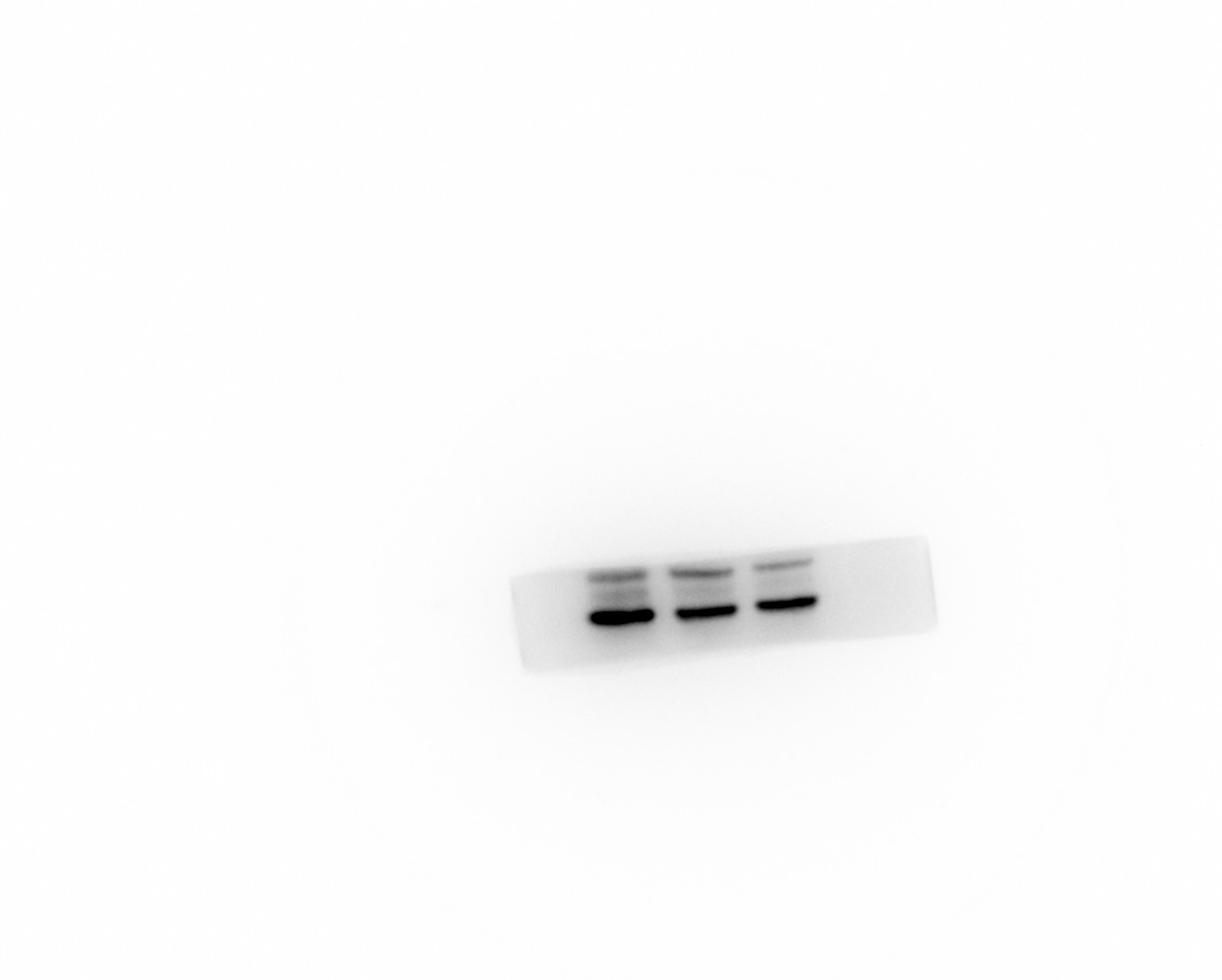


Fig7-LC3-3





Fig7-mTOR-1





Fig7-mTOR-2





Fig7-mTOR-3





Fig7-P62-1





Fig7-P62-2





Fig7-P62-3





Fig7-P70S6K-1





Fig7-P70S6K-2





Fig7-P70S6K-3





Fig7-p-mTOR-1





Fig7-p-mTOR-2





Fig7-p-mTOR-3





Fig7-P-P70S6K-1





Fig7-P-P70S6K-2





Fig7-P-P70S6K-3





Fig8b-tau46-1





Fig8b-tau46-2





Fig8b-tau46-3





Fig8b-tau181-1





Fig8b-tau181-2





Fig8b-tau181-3





Fig8b-tau205-1





Fig8b-tau205-2





Fig8b-tau205-3





Fig8b-tau404-1





Fig8b-tau404-1





Fig8b-tau404-1





Fig8c-tau46-1





Fig8c-tau46-2





Fig8c-tau46-3





Fig8c-tau181-1





Fig8c-tau181-2





Fig8c-tau181-3





Fig8c-tau205-1





Fig8c-tau205-2





Fig8c-tau205-3





Fig8c-tau404-1





Fig8c-tau404-2





Fig8c-tau404-3





Fig9-Beclin1-1





Fig9-Beclin1-2





Fig9-Beclin1-3





Fig9-GLP-1R-1

Fig9-GLP-1R-2

Fig9-GLP-1R-3

Fig9-LC3-1

Fig9-LC3-2

Fig9-LC3-3

Fig9-MAP2-1

Fig9-MAP2-2

Fig9-MAP2-3

Fig9-P62-1

Fig9-P62-2

Fig9-P62-3

Fig9-PSD95-1

Fig9-PSD95-2

Fig9-PSD95-3

Fig9-SYP-1

Fig9-SYP-2

Fig9-SYP-3
